# Sensitivity to PRIMA-1^MET^ is associated with decreased MGMT in human glioblastoma cells and glioblastoma stem cells irrespective of p53 status

**DOI:** 10.18632/oncotarget.11197

**Published:** 2016-08-11

**Authors:** Mariia Patyka, Zeinab Sharifi, Kevin Petrecca, Jose Mansure, Bertrand Jean-Claude, Siham Sabri

**Affiliations:** ^1^ Division of Experimental Medicine, Faculty of Medicine, McGill University, Montreal, Quebec, Canada; ^2^ Department of Neurology and Neurosurgery, McGill University, The Montreal Neurological Institute and Hospital, Montreal, Quebec, Canada; ^3^ Department of Urologic Oncology Research, McGill University Health Centre, Montreal, Quebec, Canada; ^4^ Department of Medicine, Division of Experimental Medicine, McGill University, The Research Institute of the McGill University Health Centre, Montreal, Quebec, Canada; ^5^ Department of Oncology, Division of Radiation Oncology, McGill University, Cancer Research Program, The Research Institute of the McGill University Health Centre, Montreal, Quebec, Canada

**Keywords:** glioblastoma, p53 mutation, MGMT, PRIMA-1^MET^/ APR-246, glioblastoma stem cells

## Abstract

Alterations of the *TP53* tumor suppressor gene occur in ~30% of primary glioblastoma (GBM) with a high frequency of missense mutations associated with the acquisition of oncogenic “gain-of-function” (GOF) mutant (mut)p53 activities. PRIMA-1^MET^/APR-246, emerged as a promising compound to rescue wild-type (wt)p53 function in different cancer types. Previous studies suggested the role of wtp53 in the negative regulation of the DNA repair protein O6-methylguanine-DNA methyltransferase (MGMT), a major determinant in resistance to therapy in GBM treatment. The potential role of MGMT in expression of p53 and the efficacy of PRIMA-1^MET^ with respect to *TP53* status and expression of MGMT in GBM remain unknown. We investigated response to PRIMA-1^MET^ of wtp53/MGMT-negative (U87MG, A172), mutp53/MGMT-positive U138, LN-18, T98/Empty vector (T98/EV) and its isogenic MGMT/shRNA gene knockdown counterpart (T98/shRNA). We show that MGMT silencing decreased expression of mutp53/GOF in T98/shRNA. PRIMA-1^MET^ further cleared T98/shRNA cells of mutp53, decreased proliferation and clonogenic potential, abrogated the G_2_ checkpoint control, increased susceptibility to apoptotic cell death, expression of GADD45A and sustained expression of phosphorylated Erk1/2. PRIMA-1^MET^ increased expression of p21 protein in U87MG and A172 and promoted senescence in U87MG cell line. Importantly, PRIMA-1^MET^ decreased relative cell numbers, disrupted the structure of neurospheres of patient-derived GBM stem cells (GSCs) and enabled activation of wtp53 with decreased expression of MGMT in MGMT-positive GSCs or decreased expression of mutp53. Our findings highlight the cell-context dependent effects of PRIMA-1^MET^ irrespective of p53 status and suggest the role of MGMT as a potential molecular target of PRIMA-1^MET^ in MGMT-positive GSCs.

## INTRODUCTION

Glioblastoma multiforme (GBM) is the most common and deadliest malignant primary brain tumor in adults [1–3]. Despite aggressive treatment involving surgery, radiation therapy (RT) and the alkylating agent temozolomide (TMZ), the prognosis for patients diagnosed with GBM remains extremely poor with a median survival of 14.6 months and only 10% of patients alive at 5 years after adjuvant chemoradiation [4–7]. The DNA repair protein O6-methylguanine-DNA methyltransferase (MGMT) removes methyl adducts from the O(6) position of guanine and, therefore, interferes with cytotoxicity of alkylating agents, including TMZ [8]. Over the last two decades, several groups identified the role of brain tumor initiating (stem) cells (BTICs) or glioma/ GBM stem cells (GSCs), as a highly tumorigenic subpopulation of cancer cells able to self-renew and generate a differentiated progeny [9, 10]. GSCs promote therapeutic resistance and drive tumor recurrence further challenging response to standard therapy [11]. In addition to the biological complexity of GBM, landmark genomic and transcriptomic studies revealed that GBM encompasses clinically relevant molecularly heterogeneous diseases classified into “proneural”, “neural”, “classical”, and “mesenchymal” subtypes [12].

The p53 tumor suppressor protein regulates cell cycle progression, DNA repair, apoptosis and senescence in response to various stress stimuli through transcriptional activation of multiple target genes, including p21^Waf1/Cip1^, the growth arrest and DNA damage 45 (GADD45A), Bax, Noxa, PUMA, KILLER/DR5, Fas, etc. [13, 14]. Alterations in *TP53* gene are reported in about 25-30% of primary GBM [15] with increased onset of *TP53* mutations in the “proneural” subtype [12, 16]. The majority of *TP53* mutations in human cancer are missense mutations that commonly occur within the DNA-binding domain of p53 resulting in disruption of p53 DNA-binding activity and impaired ability to regulate target genes and transactivate the p53 antagonist MDM2. Inhibition of MDM2-mediated mutant (mut)p53 degradation contributes within an intricate complex network to stabilization and increased expression of mutp53 protein [17, 18]. *TP53* mutations lead to abrogation of the wild-type (wt) activity of p53 and its function as a tumor suppressor gene or act as dominant negative (DN) inhibitors able to form cotetramers with co-expressed wtp53. Remarkably, *TP53* missense mutations may confer novel oncogenic properties described as mutp53 “gain-of-function” (GOF), which encompass p53 activities in the absence of co-expressed wtp53 and lead to more aggressive behavior of tumor cells such as promoting invasion, preventing apoptosis and increasing resistance to anticancer treatments [19–21]. Intriguingly, previous studies suggested the role of wtp53 in the negative regulation of MGMT levels in different human cancer cell lines including GBM [22, 23]. As a corollary, the strategy to rescue wtp53 function may concomitantly lead to decreased levels of MGMT in GBM tumors, thereby eluding resistance to alkylating agents currently used as a standard therapy in GBM treatment.

Small molecules designed to rescue wtp53 function have emerged as a potentially promising strategy to circumvent the proliferative and anti-apoptotic advantages gained through loss of p53 tumor suppressor function in different types of cancer [24–26], including gliomas [27, 28]. PRIMA-1 (p53 reactivation and induction of massive apoptosis) and its methylated and more active form PRIMA-1^MET^ (APR-246) identified by Bykov and colleagues restore mutp53 activity by promoting proper folding of the mutant protein [29, 30]. PRIMA-1^MET^ and PRIMA-1 were also shown to selectively inhibit growth and induce apoptosis in ovarian, osteosarcoma and lung cancer cell lines, harboring mutp53 *in vitro* and *in vivo* [29, 31, 32]. However, PRIMA-1^MET^ demonstrated cytotoxicity and cellular context dependency regardless of *TP53* mutational status of tumor cells in several cancer types (prostate, melanoma) [33, 34]. From a clinical point of view, PRIMA-1^MET^ is the only mutp53 reactivation compound, which showed safety, favorable pharmacokinetic profile and p53-dependent biological activity in phase I study in patients with hematologic malignancies and prostate cancer [35]. Recently, its combination with platinum-based therapy in phase Ib/II proof of concept study provided supporting evidence for the continuation of the phase II study for patients with recurrent p53 mutant high-grade serous ovarian cancer [36].

While alterations of *MGMT* and *TP53* are key determinants of GBM chemoradioresistance, understanding the potential effect of MGMT expression on p53 specifically in the context of expression of mutp53 is still lacking. Likewise, the efficacy of PRIMA-1^MET^ and its mechanism of action in GBM have not been investigated while taking into account both *TP53* status and MGMT expression levels. In this study, we investigated the potential causal relationship between MGMT and mutp53, and how MGMT may affect mutp53 GOF activities in response to PRIMA-1^MET^. To this end, we used GOF mut*TP53* [20] isogenic cell lines with at least 90% knockdown of MGMT in addition to other established GBM cell lines with different p53 status and MGMT levels. We assessed whether MGMT affects the cytotoxicity of PRIMA-1^MET^, its antiproliferative activity, its effect on clonogenic potential and the cell cycle. We also analyzed the molecular pathways underlying its cellular effects.

Given the potential role of GSCs in resistance to treatment and tumor relapse, we further investigated the effect of PRIMA-1^MET^ on patient-derived GSCs with different p53 status and MGMT levels. Our findings highlight the cell-context dependent effects of PRIMA-1^MET^ irrespective of p53 status in established GBM cell lines and GSCs. Despite their inherent genetic cell heterogeneity, we provide the first evidence that the cytotoxicity of PRIMA-1^MET^ is associated with activation of wtp53 and decreased expression of MGMT in MGMT-positive GSCs, while expression of mutp53 protein was decreased in MGMT-negative GSC line.

## RESULTS

### *In silico* analysis of the relationship between MGMT and p53 using publicly available cell lines databases

MGMT is known for its role as a DNA repair protein and loss of its expression as a result of promoter methylation has been associated with increased onset of *TP53* G:C to A:T transition mutations [37–39]. Previous studies reported the role of wtp53 in the negative regulation of MGMT levels in different human cancer cell lines [22, 23]. As a first step to investigate the relationship between MGMT and p53, we used publicly available data for their mRNA levels in the Cancer Cell Line Encyclopedia database (CCLE, http://www.broadinstitute.org/ccle) [40] and the NCI-60 cell line panel. To determine p53 status, we used information from p53 website [41, 42], COSMIC [43, 44], and literature [45, 46]. We excluded several cell lines either for misidentification, p53 null status or conflicting reports for p53 status (described in materials and methods). There was no significant correlation between mRNA levels of p53 and MGMT within all the panel of CCLE cancer cell lines originating from 24 primary sites (n = 910), neither for CCLE cancer cell lines harboring all types of alterations of *TP53* (n = 501), or only mutp53 with missense mutations (n = 355). We found a weak but significant positive correlation between mRNA levels (z-score values) of *MGMT* and *TP53* in CCLE panel of human glioma cell lines harboring wt or mutp53 (n = 42, Spearman's rho = 0.36, p value = 0.02) (Supplementary Table S1), suggesting a potential specific relationship between MGMT and p53 in primary brain tumors, compared to other types of cancer. There was a significant correlation between mRNA levels of *MGMT* and *TP53* in wtp53 glioma cell lines (n = 17, Spearman's rho = 0.55, p value = 0.024), but not between mRNA levels of *MGMT* and *TP53* in mutp53 glioma cell lines (n = 25). This may reflect the tissue and cellular specificity of mutp53 in addition to the large heterogeneity of mutp53 oncogenic proteins with either DN effect or GOF activities [47].

Expression of mRNA may not reflect protein levels, especially for genes known to be tightly regulated at the post-transcriptional level, such as *TP53* [48] and *MGMT* [49–51]. To investigate the relationship between MGMT and p53 protein expression levels, we used CellMiner database [52], which provides a web interface to access data from reverse-phase protein lysate microarrays (RPLA) in addition to other gene-based microarray platforms for NCI-60 cell lines across tumors derived from 9 different tissues. We analyzed the highest values for RPLA (log2) provided for p53 isoforms [53] and MGMT (Supplementary Table S2). There was no significant correlation between MGMT and p53 protein levels across all cell lines irrespective of their p53 status (n = 53). Analysis of the mean of RPLA protein levels strictly for cell lines harboring mutp53 revealed a strong and significant negative correlation between MGMT and mutp53 RPLA protein levels across 9 different cancer types (Pearson correlation coefficient = −0.79, p value = 0.012, n = 38). However, we could not analyze with confidence the correlation between mutp53 and MGMT RPLA protein levels within each cancer type including GBM, because of the low number of cancer cell lines with available RPLA information (Supplementary Figure S1).

### MGMT silencing decreased mutp53 protein levels in a GOF mutp53 GBM cell line

To investigate the causal link between MGMT and p53, we analyzed by Western blotting MGMT and p53 protein levels in MGMT knockdown or overexpressing isogenic GBM cell lines. We also used a panel of established GBM cell lines with known p53 status and different MGMT protein levels: MGMT-positive mutp53 GBM cell lines LN-18 (high MGMT protein levels, p53 C238S substitution) and U138 (intermediate MGMT protein levels, p53 R273H substitution) [20, 54] as well as MGMT-negative U87MG and A172 cell lines (Table 1).

**Table 1:** *TP53* status and relative p53 and MGMT protein levels in the studied human GBM cell lines

Cell line	*TP53* status	Relative p53 protein level		Relative MGMT protein level	
		Mean±SD	p-value ^a^	Mean±SD	p-value ^a^
T98/EV	M237I	1.0	-	1.0	-
T98/shRNA	M237I	0.7±0.49	<0.05	0.1±0.34	<0.05
U138	R273H	1.0	n.s.	0.6±0.13	<0.05
LN-18	C238S	0.8±0.54	<0.05	1.2±0.28	<0.05
A172	R72P heterozygous SNP	<0.1	<0.05	0	-
U87MG	Wild-type	<0.1	<0.05	0	-
U87/EV	Wild-type	<0.1	<0.05	0	-
U87/MGMT	Wild-type	<0.1	<0.05	1.6±0.18	<0.05

a Protein levels were calculated densitometrically and compared to T98/EV

SNP- single nucleotide polymorphism

We have previously used T98G, a human GBM cell line known to constitutively express high endogenous levels of MGMT and harbor GOF *TP53* mutation [20, 55] and generated stable short-hairpin (sh)RNA-mediated 90% knockdown of endogenous MGMT (T98/shRNA) and its counterpart transfected with empty vector (T98/EV) [56]. As expected, MGMT-knockdown significantly increased sensitivity of T98/shRNA to TMZ treatment in clonogenic survival assay [56]. Sequencing of *TP53* confirmed that both T98/EV and T98/shRNA cell lines possessed p53 mutation in the DNA-binding domain of the protein (M237I substitution) identical to that previously reported in T98G parental cell line (Supplementary Table S3) [41, 44]. Because of controversial reports about *TP53* status in A172, we used *TP53* sequencing and showed that A172 had R72P heterozygous single nucleotide polymorphism (SNP) in the proline-rich domain of p53, while we confirmed wtp53 status for U87MG (Supplementary Table S3) [54].

**Figure 1:**
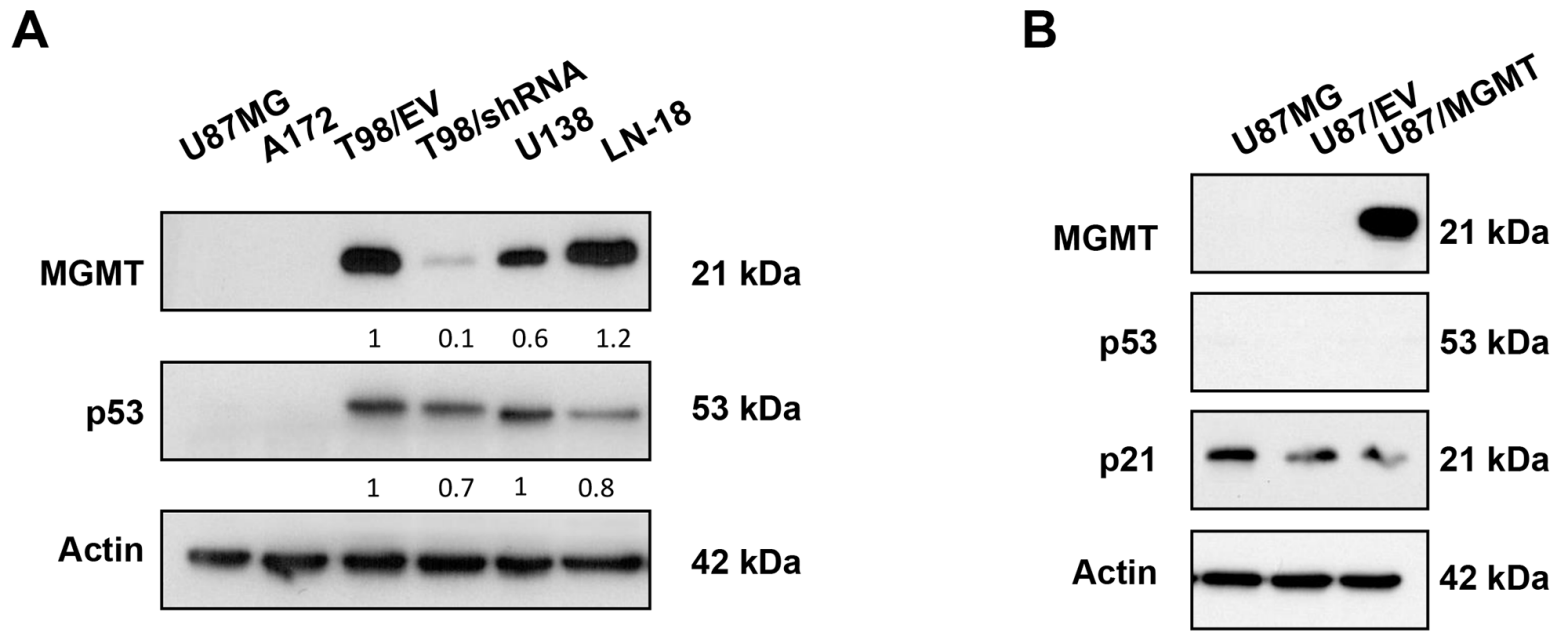
MGMT silencing decreased mutp53 protein levels in mutp53 GBM cell lines isogenic for MGMT **A.** Western blotting analysis of the effect of MGMT silencing on expression of p53. Expression of MGMT and p53 in lysates of U87MG, A172, T98G transfected with empty vector control (T98/EV) and shRNA-mediated knockdown of endogenous MGMT (T98/shRNA), as well as U138 and LN-18 GBM cell lines. **B.** Western blotting analysis of expression of MGMT, p53 and p21 in U87MG, U87/EV and U87/MGMT. Actin was used as a loading control. The density of MGMT and p53 bands was normalized to that of T98/EV.

The p53 protein is maintained at very low levels in cells with wtp53 function, while increased half-life of mutp53 protein enables its detection. Western blotting analysis using the antibody (DO-1) recognizing mutant and wtp53 showed high levels of p53 protein in mutp53 cell lines (T98/EV, T98/shRNA, U138 and LN-18) compared to wtp53 cell lines (A172, U87MG) (Figure 1A), which showed detectable basal p53 protein levels at longer exposure time (data not shown). Western blotting analysis of p21 confirmed the lack of p21 expression in mutp53 cell lines and its basal expression in U87MG and A172 cell lines (data not shown). Interestingly, densitometric analysis showed that knockdown of MGMT in T98/shRNA cell line (>90%) was associated with a significant decrease of mutp53 protein levels by 35±4.9% (p value < 0.05) (Figure 1A, Table 1). Levels of p53 in LN-18 cells were 23±5.4% lower than in T98/EV (p value < 0.05). Overexpression of MGMT (U87/MGMT) did not affect wtp53 or p21 protein levels, compared to its MGMT-negative counterpart empty vector (U87/EV) control (Figure 1B). Hence, MGMT silencing was associated with decreased mutp53 protein levels in a GOF mutp53 GBM cell line. Conversely, overexpression of MGMT did not affect p53 levels in wtp53 GBM cells, suggesting that the relationship between MGMT and p53 is restricted to GOF mutp53 context.

### PRIMA-1^MET^ induces cytotoxic effects in GBM cell lines irrespective of p53 status

We used PRIMA-1^MET^ to test the functional consequences of down-regulation of MGMT expression levels in our MGMT isogenic cell lines with GOF mutp53 background. We assessed cytotoxic effects of PRIMA-1^MET^ (24-hour treatment) in GBM cell lines based on MGMT expression and *TP53* status. First, to test the viability of GBM cell lines *in vitro* we treated T98/EV, T98/shRNA, U138, LN-18, U87MG and A172 cell lines with 25, 50, 75 or 100 μM PRIMA-1^MET^ for 24 hours, then cells were kept in drug-free medium for 24 hours (48-hour time point) or 48 hours (72-hour time point). We examined the relative cell number (percentage relative to DMSO control) and viable cell number (% relative to total cell number in each experimental condition) at each time point (24, 48 or 72 hours) using trypan blue exclusion assay and automated cell counting.

**Figure 2:**
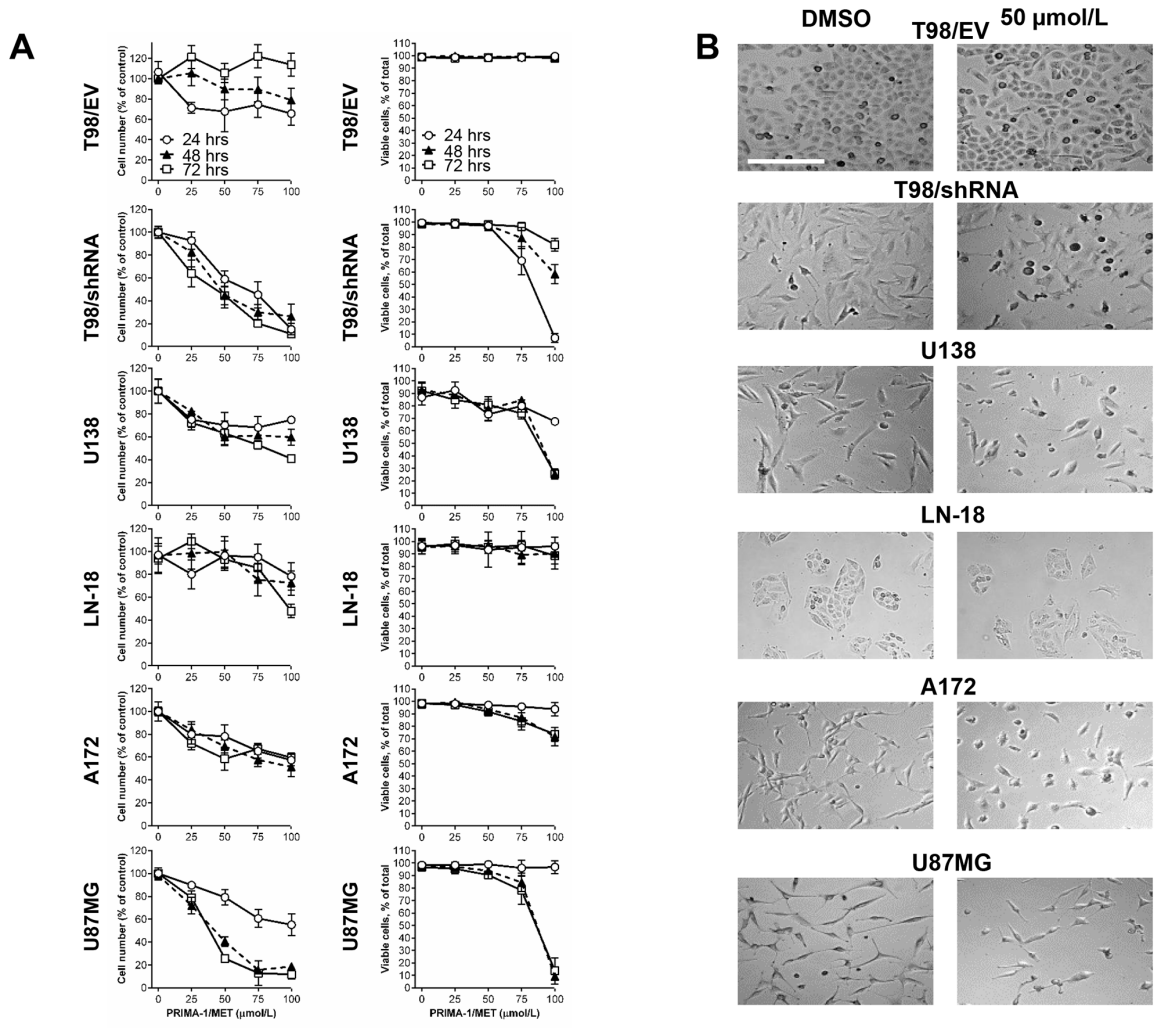
PRIMA-1^MET^ reduced relative cell number of GBM cell lines irrespective of p53 status **A.** Analysis of the cytotoxic effect of PRIMA-1^MET^ on T98/EV, T98/shRNA, U138, LN-18, A172 and U87MG GBM cell lines using trypan blue exclusion assay and automated cell counting to determine the percentage of relative number of cells in PRIMA-1^MET^-treated conditions relative to DMSO control at each time point (24, 48 or 72 hours following initiation of a 24-hour treatment with PRIMA-1^MET^) (left) and the ratio of viable cells (% relative to total cell number in each experimental condition) (right) in the indicated cell lines. Data on graphs represent the mean values ± SD and are representative of at least three independent experiments. **B.** Representative micrographs of GBM cells (original magnification 100X) treated with PRIMA-1^MET^ (50 μM, 24 hours) or DMSO control. Scale bar = 250 μm.

The results showed that PRIMA-1^MET^ at 25 μM reduced the relative cell number in T98/EV by 28.8±5.3% at 24 hours, but higher doses were not more effective (Figure 2A and Table 2). In addition, following drug removal, the cell number was completely restored at 48 and 72-hour time points and was not reduced relative to their respective DMSO controls. By contrast, in T98/shRNA PRIMA-1^MET^ reduced relative cell number in a time and dose-dependent manner (e.g., by 55.5±7.9% and 89.1±1.3% at 50 μM and 100 μM, respectively, at 72-hour time point). The relative cell number decrease in T98/shRNA following 100 μM was significantly greater, compared to that in T98/EV, at all time points (Table 3). In U138 cell line, PRIMA-1^MET^ significantly decreased the relative cell number by 37±10.7% at 50 μM and by 59.1±3.1% at 100 μM at 72-hour time point, while in LN-18 the relative cell number was significantly decreased at 100 μM (by 52.1±5.8%), but not at 50 μM (Figure 2A and Table 2). Treatment with PRIMA-1^MET^ at 50 μM and 100 μM significantly decreased the relative cell number U87MG cell line by 74.4±3.4% and 88.3±3.9%, respectively, at 72 hours, while in A172 similar doses decreased the relative cell number by 41.5±9.96% and 40.3±4%, respectively.

**Table 2:** Relative cell number and viable cells (%) in GBM cell lines treated with a range of PRIMA-1^MET^ doses

PRIMA-1^MET^, μM	24 hours		48 hours		72 hours	
	Cell number, %^a^	p-value^b^	Cell number, %^a^	p-value^b^	Cell number, %^a^	p-value^b^
	**T98/EV**					
0	107±9.99	-	100.2±2.3	-	100±5.03	-
25	71.2±5.25	0.0029	105±12.6	n.s.	121±11.5	0.03
50	67.6±19.7	0.0062	89.5±10.1	n.s.	106±9.56	n.s.
75	74.5±12.6	0.0097	89.5±12.1	n.s.	122±11.5	0.009
100	65.6±11.3	0.0017	78.9±11.5	n.s.	113.7±11.2	n.s.
	**T98/shRNA**					
0	100±5.4	-	100±4.98	-	100±1.91	-
25	92.6±7.7	n.s.	82.5±12.8	0.02	63.9±11.7	< 0.0001
50	59.0±6.95	< 0.0001	44.9±11.4	< 0.0001	44.5±7.87	< 0.0001
75	45.2±11.4	< 0.0001	29.9±6.79	< 0.0001	20.2±1.97	< 0.0001
100	15.0±5.0	< 0.0001	26.3±10.7	< 0.0001	11.0±1.3	< 0.0001
	**U138**					
0	100±1.8	-	100±10.7	-	100±10.4	-
25	75.1±7.1	0.01	82.4±1.05	0.017	72.3±6.02	< 0.0001
50	70.2±11.3	0.003	59.98±6.7	< 0.0001	63.1±10.7	< 0.0001
75	68.2±9.7	0.001	61.2±1.5	0.0002	52.9±3.4	< 0.0001
100	74.8±1.6	0.017	59.7±7.0	0.0002	40.9±3.1	< 0.0001
	**LN-18**					
0	97±7.97	-	96.8±14.96	-	93.9±13.2	-
25	80.1±12.7	< 0.0001	98.5±13.7	n.s.	108.9±6.5	< 0.0001
50	96.4±12.6	n.s.	99.8±13.2	n.s.	93.1±7.99	n.s.
75	95.1±11.4	n.s.	75.3±14.3	< 0.0001	85.7±11.6	0.006
100	78.3±12.1	< 0.0001	72.5±10.6	< 0.0001	47.9±5.8	< 0.0001
	**A172**					
0	100±8.2	-	100±8.6	-	100±3.7	
25	79.95±8.6	0.002	83.9±6.8	0.004	72.2±5.6	< 0.0001
50	78.2±9.97	0.0004	69.4±5.8	< 0.0001	58.5±9.96	< 0.0001
75	65.3±5.9	< 0.0001	57.7±6.0	< 0.0001	67.3±4.5	< 0.0001
100	57.4±5.0	< 0.0001	51.4±8.6	< 0.0001	59.7±4.0	< 0.0001
	**U87MG**					
0	100±2.8	-	98.2±4.1	-	100±5.0	-
25	89.8±2.1	0.001	71.95±7.2	< 0.0001	78.7±6.1	0.009
50	79.2±6.8	0.0005	40.5±4.3	< 0.0001	25.6±3.4	< 0.0001
75	60.7±7.7	< 0.0001	15.6±2.3	< 0.0001	12.97±10.79	< 0.0001
100	55.2 ±9.7	< 0.0001	18.7±2.0	< 0.0001	11.7±3.9	< 0.0001

PRIMA-1^MET^, μM	24 hours		48 hours		72 hours	
	Viable cells,%^a^	p-value^b^	Viable cells, %^a^	p-value^b^	Viable cells, %^a^	p-value^b^
	**T98/EV**					
0	99±1.4	-	99.3±1.0	-	98.9±0.3	-
25	98.8±1.6	n.s.	99.8±0.5	n.s.	97.99±1.6	n.s.
50	98.3±2.4	n.s.	99±0.8	n.s.	98.7±1.2	n.s.
75	98.6±1.3	n.s.	99.5±0.6	n.s.	99.1±1.0	n.s.
100	99.6±0.9	n.s.	98.5±1.7	n.s.	98.1±1.9	n.s.
	**T98/shRNA**					
0	99.5±1.0	-	98.4±0.9	-	98.5±0.8	-
25	98.5±1.7	n.s.	98±1.5	n.s.	99.2±0.98	n.s.
50	97±3.9	n.s.	97.3±1.9	n.s.	98.03±2.4	n.s.
75	69±11.3	< 0.0001	87.3±8.4	0.005	96.4±3.4	n.s.
100	7±3.6	< 0.0001	58.3±7.8	< 0.0001	81.8±5.02	< 0.0001
	**U138**					
0	86.9±6.2	-	92.8±6.5	-	92.1±6.3	-
25	92.5±6.5	n.s.	88.8±0.4	n.s.	84.8±6.6	n.s.
50	73.3±5	0.001	77.6±9.6	0.01	81±4.3	n.s.
75	79.95±5	n.s.	84.6±0.7	n.s.	73.9±4.2	< 0.0001
100	67.5±1.9	0.001	25.2±3.7	< 0.0001	25.4±4.1	< 0.0001
	**LN-18**					
0	96.3±6.2	-	95.1±5.2	-	95.7±5.4	-
25	96.8±6.6	n.s.	96.3±4.6	n.s.	98±3.1	n.s.
50	93.3±14.1	n.s.	96.9±3.2	n.s.	95.5±5.1	n.s.
75	95.2±12.9	n.s.	89.2±8	< 0.0001	96.7±4.3	n.s.
100	96.1±7.1	n.s.	90.3±8.6	0.0007	88.5±10.6	< 0.0001
	**A172**					
0	98.3±1.4	-	97.5±1.6	-	98.3±0.9	-
25	98±0.6	n.s.	99.4±0.8	n.s.	97.2±1.3	n.s.
50	97.1±2.2	n.s.	93.6±3.8	n.s.	91.8±3.6	0.031
75	95.7±2.5	n.s.	86.9±7.2	0.0055	83.9±6.9	< 0.0001
100	93.7±5.3	0.027	71.7±7.3	< 0.0001	73.6±5.7	< 0.0001
	**U87MG**					
0	98.4±1.5	-	96.9±2	-	96.8±3.3	-
25	98.4±1.3	n.s.	97.1±3.6	n.s.	95.4±4.1	n.s.
50	99.03±0.8	n.s.	93.8±6.5	n.s.	90.4±0.2	n.s.
75	96.1±6.1	n.s.	84.5±7.4	0.0027	78.1±11.3	0.0005
100	96.8±5.3	n.s.	9.2±2.0	< 0.0001	13.7±10.5	< 0.0001

a Mean ± SD

b Compared to DMSO control at the corresponding time point

n.s. – not significant

**Table 3:** Cell number (%) in PRIMA-1^MET^-treated conditions (100 μM)

Cell line	24 hours		48 hours		72 hours	
	Cell number, %^a^	p-value^b^	Cell number, %^a^	p-value^b^	Cell number, %^a^	p-value^b^
T98/EV	65.6±11.3	-	78.9±11.5	-	113.7±11.2	-
T98/shRNA	15.0±5.0	< 0.0001	26.3±10.7	< 0.0001	11.0±1.3	< 0.0001
U138	74.8±1.6	n.s.	59.7±7.0	n.s.	40.9±3.1	< 0.0001
LN-18	78.3±12.1	n.s.	72.5±10.6	n.s.	47.9±5.8	< 0.0001
A172	57.4±5.0	n.s.	51.4±8.6	0.002	59.7±4.0	< 0.0001
U87MG	55.2 ±9.7	n.s.	18.7±2.0	< 0.0001	11.7±3.9	< 0.0001

a Mean ± SD (relative to DMSO control)

b Compared to T98/EV at the corresponding time point

n.s. – not significant

Decreased viability (% of viable cells) was dose-dependent for T98/shRNA, U87MG, A172 and U138 cell lines reaching 18.2±5%, 86.3±10.5%, 26.4±5.7% and 74.6±4.1% decrease, respectively, and only 11.5±10.6% decrease for LN-18 for PRIMA-1^MET^ at 100 μM, 72 hours following treatment (p value < 0.01) (Figure 2A and Table 2). By contrast, PRIMA-1^MET^ did not induce decreased cell viability in T98/EV up to 100 μM during 72-hour time course. Thus, PRIMA-1^MET^ induced cytotoxicity mostly through reducing cell number in T98/shRNA, U138, LN-18, A172 and U87MG cell lines, but not in T98/EV.

Consistent with the quantitative results of the viability assay, the morphological examination showed the predominance of a rounded shape, the presence of sparse and floating cells in T98/shRNA, U87MG and U138, but not T98/EV, A172 or LN-18 cells treated with PRIMA-1^MET^, compared to their respective controls (Figure 2B). Taken together, our results show that PRIMA-1^MET^ preferentially induced time and dose-dependent cytotoxicity mostly through reduced cell number irrespective of p53 status. With the exception of A172, MGMT-negative or low MGMT levels GBM cell lines T98/shRNA, U87MG and U138 were the most sensitive to PRIMA-1^MET^ at all time points.

### PRIMA-1^MET^ decreased proliferation and clonogenic potential irrespective of p53 status in GBM cell lines

**Figure 3:**
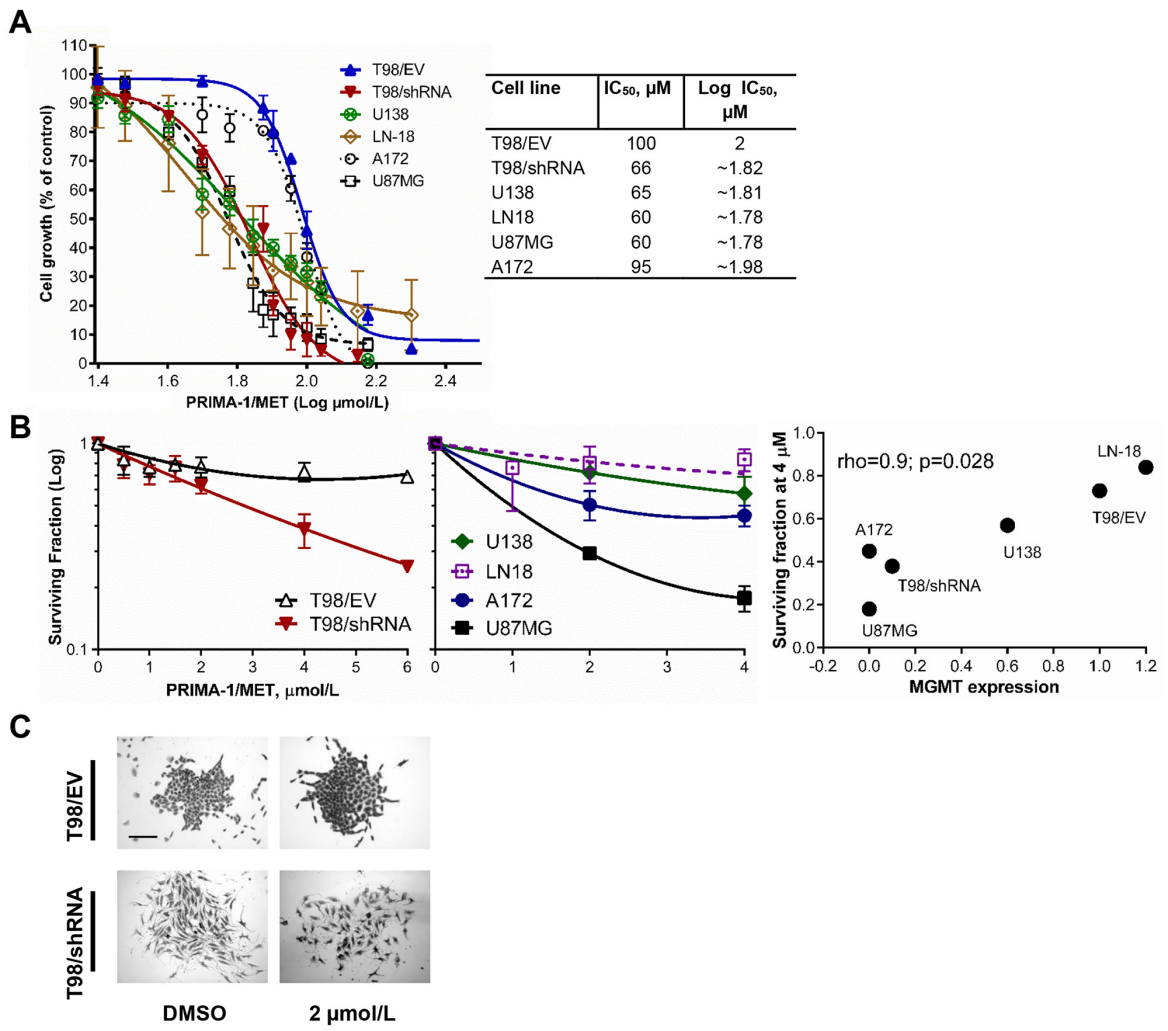
PRIMA-1^MET^ decreased proliferation and clonogenic potential of GBM cell lines with different MGMT levels and p53 status **A.** Growth-inhibitory effects examined by MTT assay after incubation of T98/EV, T98/shRNA, U138, LN-18, A172 and U87MG GBM cell lines for 24 hours with increasing doses of PRIMA-1^MET^ (10-200 μM) and additional 24 hours in a drug-free medium. Concentration of PRIMA-1^MET^ is on a log_10_ scale. Graphs represent mean values ± SD from at least three independent experiments performed in triplicate. The resulting IC_50_ values are shown in the table. **B.** Colony formation assay results for T98/EV, T98/shRNA (left), LN-18, U138, A172 and U87MG (center) GBM cell lines - the number of colonies (more than 50 cells) was counted and surviving fraction was calculated 8-14 days after treatment with the indicated concentrations of PRIMA-1^MET^ for 24 hours and further incubation in a drug-free medium. Surviving fraction (Y axis, log-scale) was normalized to plating efficiency of the corresponding DMSO controls. Results are means ± SD for at least three independent experiments performed in triplicate. Correlation between MGMT protein levels (from Table 1) and surviving fraction of T98/EV, T98/shRNA, LN-18, U138, A172 and U87MG GBM cell lines (right) treated with 4 μM PRIMA-1^MET^. **C.** Representative micrographs of T98/EV and T98/shRNA cell colonies stained with methylene blue 7 days following 24-hour treatment with 2 μM PRIMA-1^MET^ (original magnification 100X). Scale bar = 250 μm.

We further investigated the effect of PRIMA-1^MET^ on proliferation of GBM cell lines using the MTT proliferation assay in GBM cells treated with doses of PRIMA-1^MET^ ranging between 10 and 200 μM. Results of the MTT assay were consistent with viability analysis using the trypan blue exclusion assay. As shown in Figure 3A, PRIMA-1^MET^ at 50 μM (corresponding to 1.7 μM on the log scale for the IC_50_ sigmoidal dose-response curve) did not alter proliferation of T98/EV, but inhibited proliferation of T98/shRNA, U138, LN-18, U87MG and A172 cell lines by 28%, 42%, 48%, 30% and 14% (p value < 0.0001), respectively. The IC_50_ for each cell line was as follows: T98/EV - 100 μM, T98/shRNA - 66 μM, U87MG – 60 μM, A172 - 95 μM, U138 – 65 μM, LN-18 – 60 μM. The sensitivity of wtp53 U87MG cells to PRIMA-1^MET^, which is in the same range as mutp53 T98/shRNA or U138 suggests that this compound can possibly decrease cell growth independently of p53 status in GBM cells.

To further explore the cytotoxic effects induced by PRIMA-1^MET^, we carried out a clonogenic assay to analyze the colony formation ability following treatment of GBM cells with PRIMA-1^MET^. All cell lines failed to form any colonies at doses higher than 6 μM, suggesting that exposure to PRIMA-1^MET^ for only 24 hours induced long-term cytotoxic effects at lower concentrations than IC_50_, irrespective of p53 status.The colony-forming ability of T98/EV cells after exposure to PRIMA-1^MET^ at 4 μM was minimally affected and showed a reduction of ~27±7% (p value < 0.0001) (Figure 3B). T98/shRNA exhibited a stronger dose-dependent inhibition ~61.7±7.2% at 4 μM (p value < 0.0001). The significant difference in response of T98/shRNA, compared to T98/EV, was detected at a concentration as low as 2 μM (p value < 0.005) and became more drastic with higher concentrations (p value < 0.0001 at 4 μM). The colony formation ability of LN-18 was not significantly decreased (~16.2±10.2% decrease) at 4 μM, but was suppressed by ~42.8±11.7%, ~57.1±4.7% and ~82.2±2.5% in U138, A172 and U87MG, respectively (p value < 0.001). MGMT protein levels in the tested GBM cell lines significantly correlated with their respective surviving fraction following exposure to 4 μM PRIMA-1^MET^ (n = 6, Spearman's rho = 0.9, p value = 0.028) (Figure 3B). Of note, even at a concentration as low as 2 μM, PRIMA-1^MET^ induced spindle-shaped cell morphology and dispersed colonies in T98/shRNA cell line, compared to tight colonies in the DMSO control (Figure 3C).

Taken together, our findings suggest that PRIMA-1^MET^ inhibits proliferation and colony-forming potential of GBM cells independently of their p53 status. MGMT silencing caused decreased expression of mutp53 in T98/shRNA cells, which possibly contributes to sensitizing these cells to the anti-proliferative effects of PRIMA-1^MET^. High levels of MGMT correlate with increased resistance to PRIMA-1^MET^, while its low levels correlate with increased sensitivity to PRIMA-1^MET^ through long-term effects in GBM cell lines irrespective of their p53 status.

### PRIMA-1^MET^–induced G2/M checkpoint abrogation is associated with MGMT silencing

**Figure 4:**
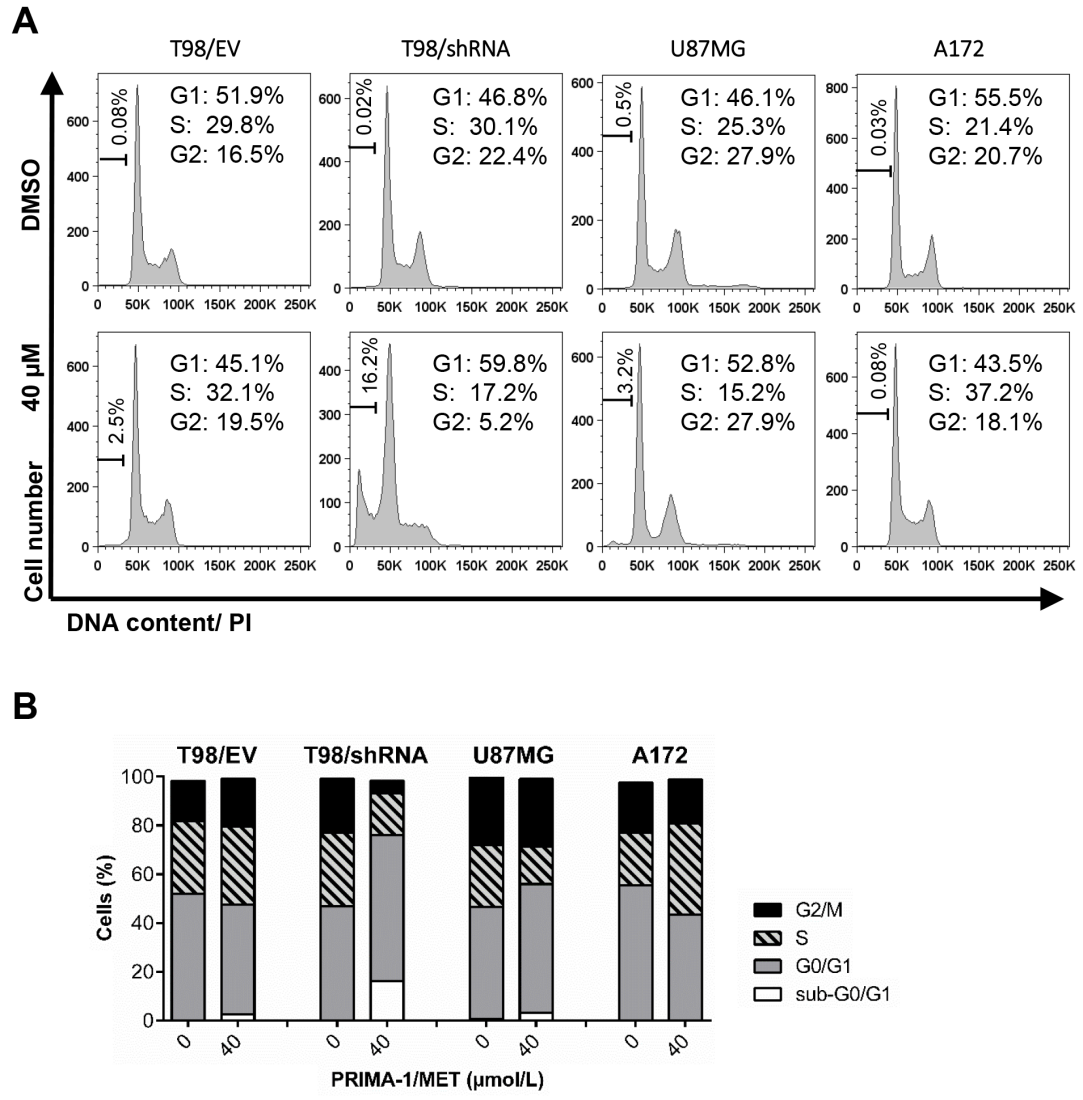
PRIMA-1^MET^ induced changes in cell cycle progression in GBM cells with silenced MGMT **A.** Representative histogram plots of cell cycle distribution in T98/EV, T98/shRNA, U87MG and A172 GBM cell lines stained with propidium iodide (PI) at 24 hours following initiation of treatment with 40 μM PRIMA-1^MET^ or DMSO and analyzed by flow cytometry. **B.** Bar graphs illustrate results of cell cycle analysis shown in (A), indicating the percentage of cells in sub-G0/G1, G0/G1, S, and G2/M cell cycle phases after treatment with 40 μM PRIMA-1^MET^ or DMSO.

To further investigate the cell type-specific effects of PRIMA-1^MET^, we tested whether the anti-proliferative effect of PRIMA-1^MET^ was mediated by changes in cell cycle progression. GBM cells were treated with a range of PRIMA-1^MET^ concentrations or DMSO and cell cycle distribution was analyzed with propidium iodide staining using flow cytometry (Figure 4). Quantification of the percentage of cells in different cell cycle phases indicated that treatment with 25 μM PRIMA-1^MET^ for 24 hours induced a significant increase in a percentage of cells in G2/M phase (from 23.1% to 33.5%) in T98/shRNA compared to DMSO control (data not shown), while 40 μM completely abrogated G2/M checkpoint (Figure 4A and 4B). By contrast, no change was observed after exposure to PRIMA-1^MET^ in T98/EV, confirming the results of cell viability and proliferation assays. In A172, 40 μM PRIMA-1^MET^ delayed progression through the S-phase (from 21.4% to 37.2%), while in U87MG the cell cycle arrest in G1-phase was detected (from 46.1% to 52.8%) with concomitant decrease in the S-phase. Quantification of cells with sub-G0/G1 DNA content showed that 40 μM PRIMA-1^MET^ induced accumulation of cells in the sub-G0/G1 phase of cell cycle in T98/shRNA (from 0.02% to 16.2%) and to a much less extent in T98/EV and U87MG. Treatment with PRIMA-1^MET^ did not induce changes in sub-G0/G1 population in A172 cells.

### PRIMA-1^MET^ induces dose-dependent decrease of mutp53 protein, increased PARP-1 cleavage and expression of GADD45A in the context of MGMT silencing

**Figure 5:**
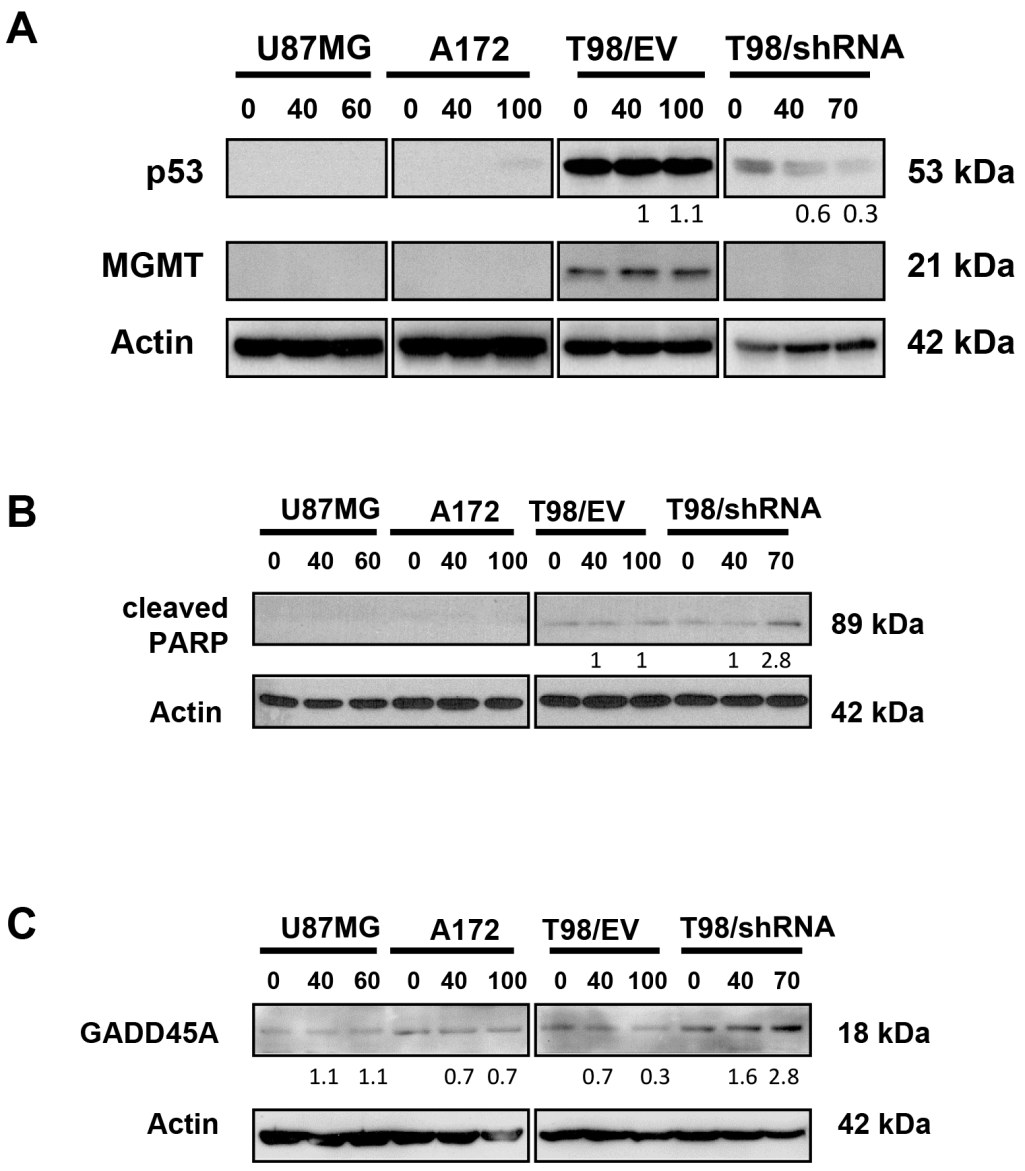
PRIMA-1^MET^ decreased expression of mutp53 and increased cleaved PARP-1 and GADD45A in GBM cells with MGMT knockdown Western blotting analysis of expression of MGMT and p53 **(A)** cleaved form of PARP-1 (89 kDa) **(B)** and GADD45A **(C)** in U87MG, A172, T98/EV and T98/shRNA GBM cell lines following 24-hour treatment with 40 μM (common dose) or the concentration corresponding to IC_50_ value of PRIMA-1^MET^ in each cell line. Actin was used as a loading control. The density of the bands was normalized to that of DMSO controls (taken as 100%).

To investigate the molecular effects of PRIMA-1^MET^, T98/EV, T98/shRNA, U87MG and A172 cells were treated using their respective IC_50_ values for 24 hours, lysed and assessed for p53 and MGMT expression using Western blotting. We confirmed decreased p53 levels following MGMT knockdown in T98/shRNA (DMSO control) compared to T98/EV (Figure 5A). Strikingly, PRIMA-1^MET^ further suppressed p53 expression in T98/shRNA in a dose-dependent manner. By contrast, PRIMA-1^MET^ treatment did not affect p53 or MGMT expression levels in T98/EV, U87MG or A172 cell lines.

Cleavage of poly(ADP-ribose) polymerase (PARP-1) into fragments of 89 and 24 kDa is a hallmark of apoptosis. Cleaved PARP-1 fragment (89 kDa) was detected by Western blotting in T98/shRNA cells treated with 70 μM PRIMA-1^MET^, but not in other cell lines (Figure 5B), which is in accordance with cell cycle analysis showing the accumulation of T98/shRNA cells in the sub-G0/G1 phase of cell cycle in T98/shRNA.

*GADD45A*, a DNA damage inducible gene involved in cell cycle arrest and apoptosis is regulated through p53-dependent and independent mechanisms. Interestingly, expression of GADD45A protein increased in T98/shRNA compared to T98/EV. This increase was more pronounced following exposure to PRIMA-1^MET^ (Figure 5C) and was maintained up to 48 hours (data not shown). Thus, abrogation of G2 checkpoint and increased sub-G0/G1 cell population detected after PRIMA-1^MET^ treatment is associated with suppression of mutp53 protein expression, increased expression of GADD45A and cleaved PARP-1 in T98/shRNA cells.

### PRIMA-1^MET^ induces senescent phenotype in wtp53 U87MG MGMT-negative GBM cell line

**Figure 6:**
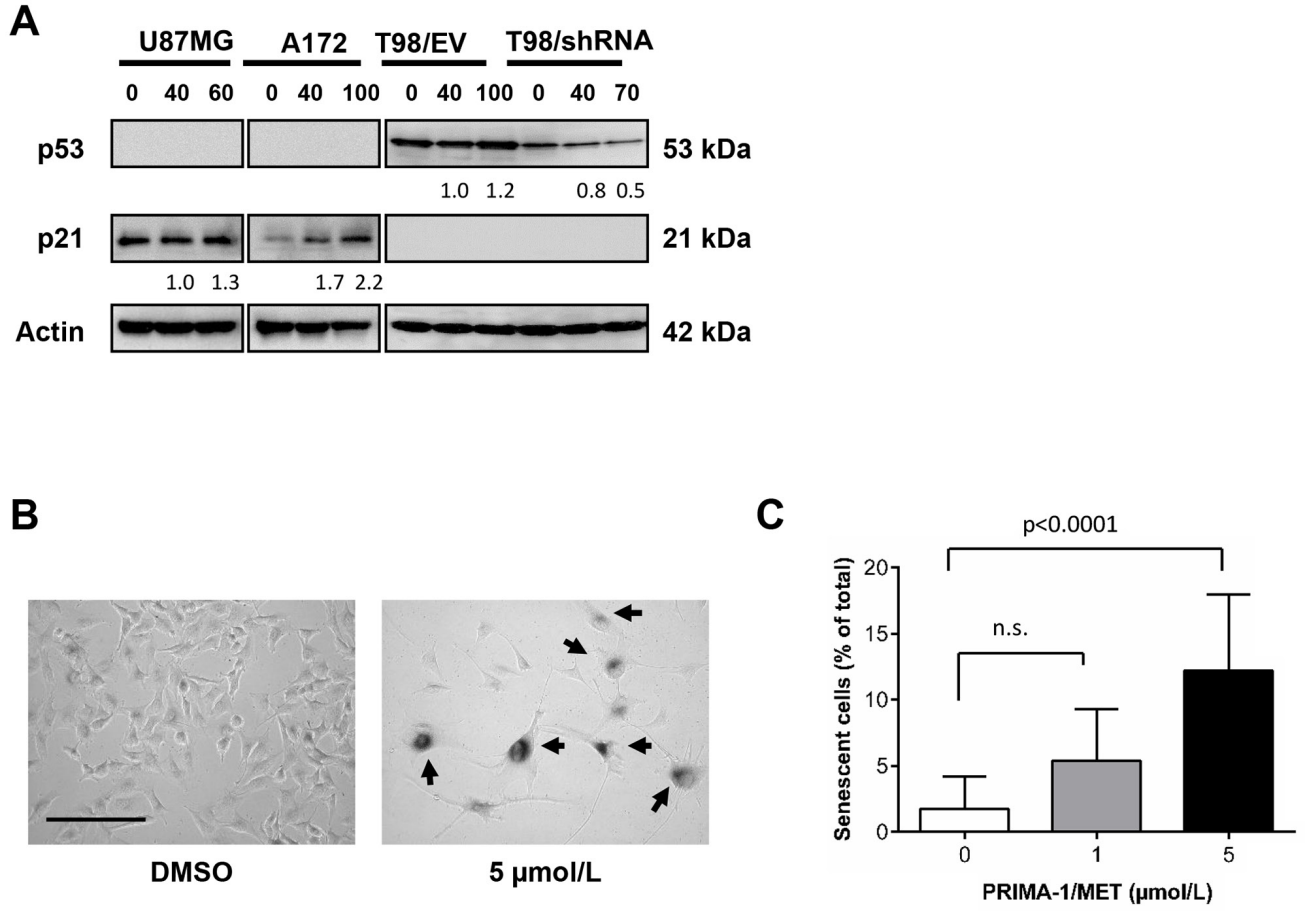
PRIMA-1^MET^ treatment increased p21 and senescent phenotype in wtp53 MGMT-negative GBM cells **A.** Western blotting analysis of expression of p53 and p21 in U87MG, A172, T98/EV and T98/shRNA GBM cell lines following 24-hour treatment with 40 μM (common dose) or the concentration corresponding to IC_50_ value of PRIMA-1^MET^ in each cell line. Actin was used as a loading control. The density of the bands was normalized to that of DMSO controls (taken as 100%). **B.** Representative micrographs of senescence-associated β-galactosidase (SA-β-gal)-positive U87MG cells 6 days after the initiation of treatment with 5 μM PRIMA-1^MET^ (original magnification 200X). Arrows show senescent cells. Scale bar = 200 μm. **C.** Percentage of SA-β-gal-positive U87MG cells 6 days after the initiation of treatment with 1 or 5 μM PRIMA-1^MET^. Results are means ± SD; total number of cells counted in each condition > 400. P-value for each condition compared to DMSO control is shown; n.s. – not significant.

To determine the effect of PRIMA-1^MET^ on one of the main p53 targets - cyclin-dependent kinase inhibitor p21, cells were treated by PRIMA-1^MET^ and lysed to assess p21 protein expression by Western blotting. PRIMA-1^MET^ was unable to induce p21 transactivation in GBM cell lines T98/EV and T98/shRNA harboring mutp53 (Figure 6A). By contrast, cell lines possessing wtp53, U87MG and A172, showed upregulation of p21 expression upon PRIMA-1^MET^ treatment. Furthermore, U87MG cells treated with as low as 1 μM of PRIMA-1^MET^ exhibited senescent phenotype (Figure 6B) as visualized by a positive staining for β-Galactosidase with higher frequency than DMSO control (p value < 0.0001) (Figure 6C), while doses above 10 μM led to a massive cell death. By contrast, PRIMA-1^MET^ did not induce senescence in A172, despite elevated p21 levels, or in T98/EV and T98/shRNA (< 0.001% of senescent cells).

### PRIMA-1^MET^ induces sustained activation of phosphorylated forms of Erk1/2, which is associated with MGMT silencing

Activation of extracellular signal-regulated kinase 1/2 (Erk1/2) has been involved in growth, proliferation, regulation of p53 among other transcription factors, but also in apoptosis [57]. Given the inhibition of proliferation and induction of apoptosis observed following MGMT silencing with PRIMA-1^MET^ treatment, we used Western blotting to assess phosphorylation status (p-Erk1/2) relative to total Erk1/2 as a readout of its activation in U87MG, A172, T98/EV and T98/shRNA cells.

**Figure 7:**
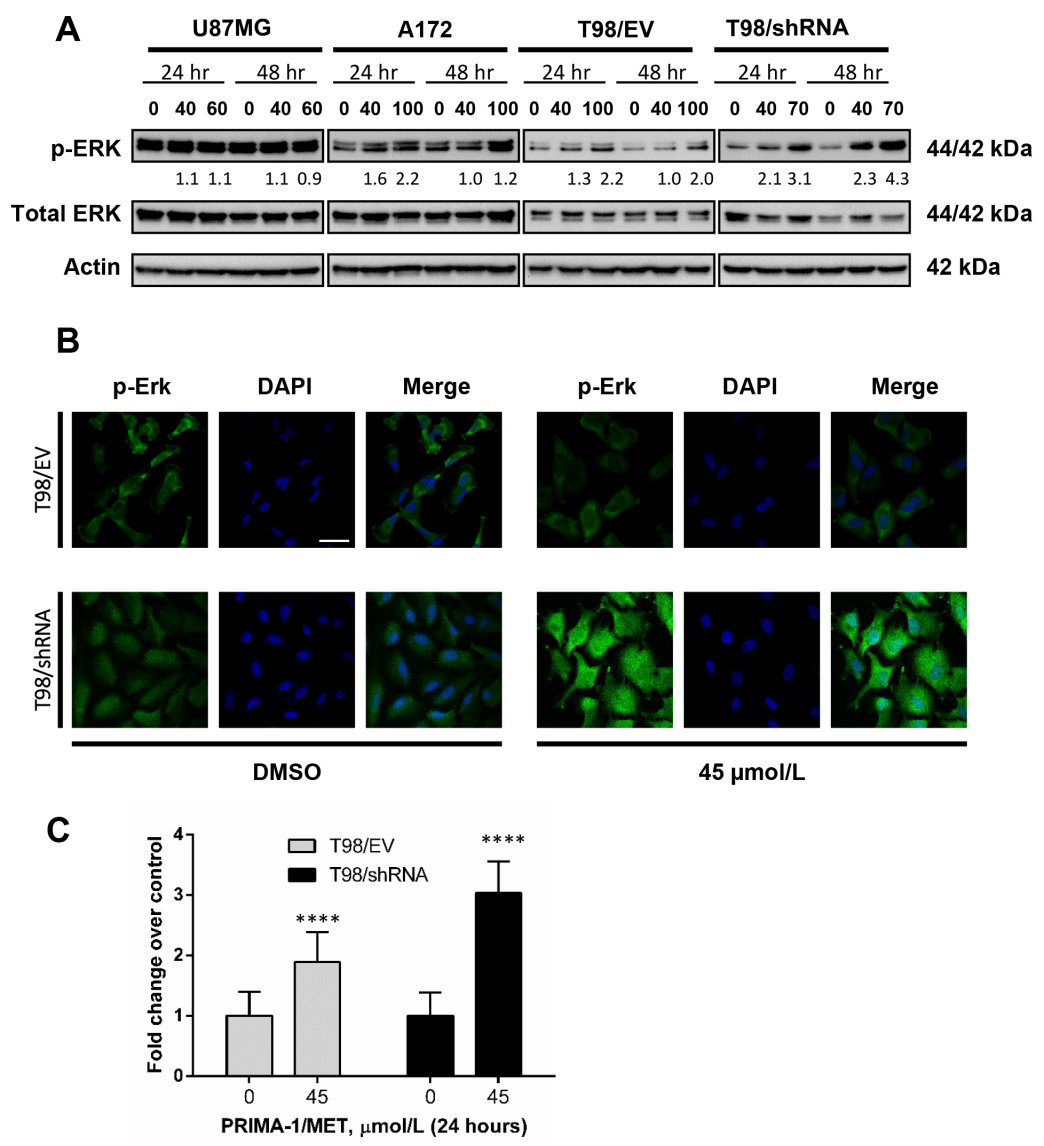
PRIMA-1^MET^ modulated expression and distribution of phosphorylated forms of Erk1/2 in GBM cell lines **A.** Western blot analysis showing expression of phosphorylated forms of Erk1/2 (Thr202/Tyr204) in U87MG, A172, T98/EV and T98/shRNA GBM cell lines at 24 or 48 hours following initiation of PRIMA-1^MET^ treatment with 40 μM (common dose) or the concentration corresponding to IC_50_ value of PRIMA-1^MET^ in each cell line (actin used as a loading control). The density of the bands was normalized to that of DMSO controls (taken as 100%). **B.** Immunofluorescence staining and confocal microscopy analysis of T98/EV and T98/shRNA cells to assess intensity and localization of the phosphorylated forms of Erk1/2 at 24 hours following initiation of treatment with 45 μM PRIMA-1^MET^ (45 μM is ~ IC_20_ for T98/shRNA and < IC_10_ for T98/EV) (original magnification 400X). Scale bar = 50 μm. **C.** Fold-changes in expression of the phosphorylated forms of Erk1/2 in T98/EV and T98/shRNA GBM cells at 24 hours following initiation of treatment with 45 μM PRIMA-1^MET^ as assessed by immunofluorescent staining using ImageJ software. Results are means ± SD for representative of at least three independent experiments. Total number of cells analyzed in each condition of experiment > 40 cells. ****, statistically significant difference (p < 0.0001) compared to DMSO control.

In U87MG, A172 and T98/EV cells, total levels of Erk1/2 were unchanged with PRIMA-1^MET^ treatment over 24 hours. Interestingly, treatment with PRIMA-1^MET^ induced drastic increase of p-Erk1/2 in T98/shRNA cells (Figure 7A), which persisted up to 48 hours following treatment initiation. The expression of p-Erk1/2 was increased to a much less extent in T98/EV and A172 cells, but not in U87MG. Furthermore, fluorescence microscopy showed that PRIMA-1^MET^ did not affect p-Erk1/2 localization in the perinuclear region of T98/EV cells. By contrast, PRIMA-1^MET^ induced a substantial increase in p-Erk1/2 levels and its cytoplasmic localization in T98/shRNA compared to control (Figure 7B and 7C).

### PRIMA-1^MET^ induces cytotoxic effects in GSCs irrespective of p53 status

**Figure 8:**
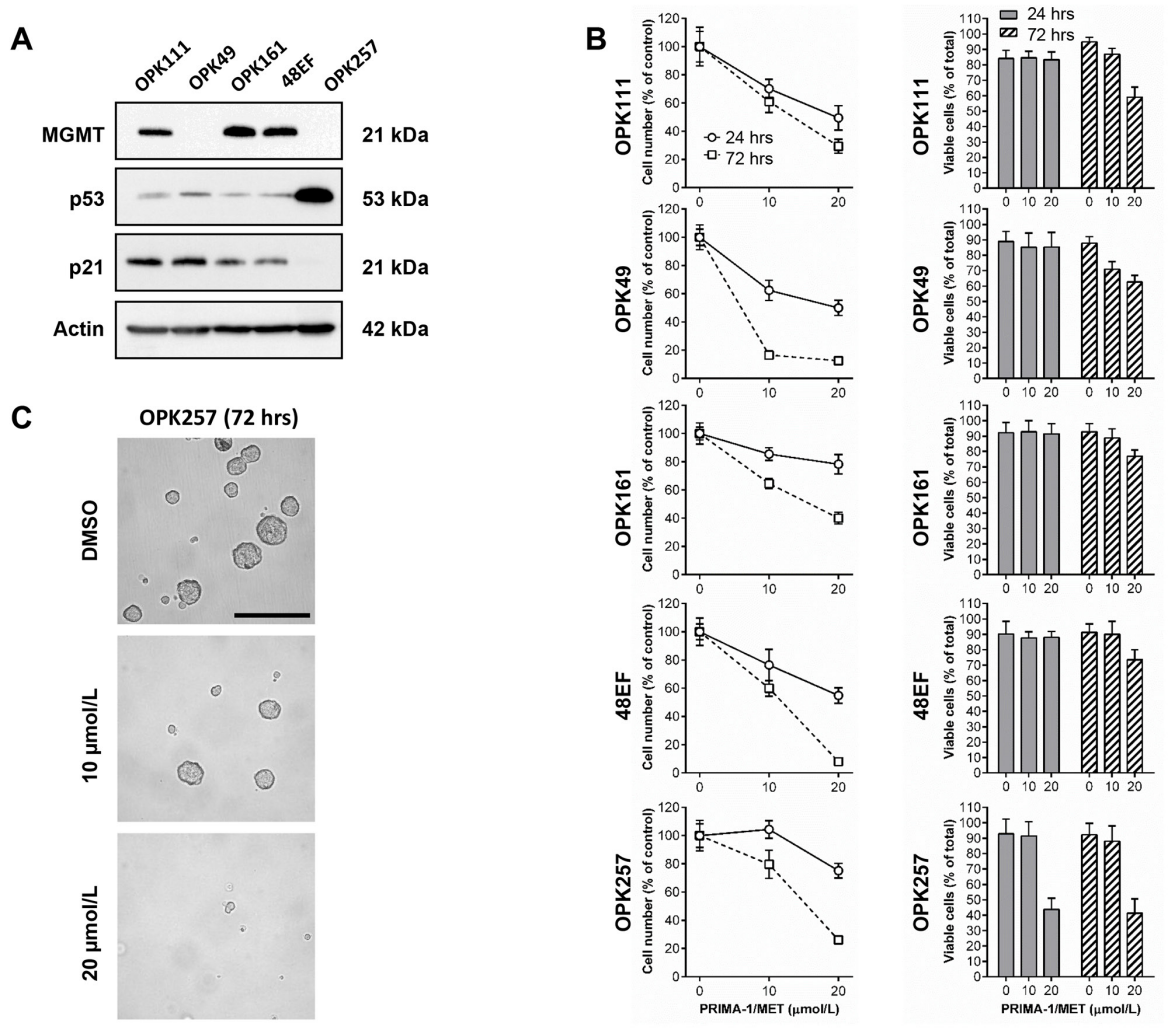
PRIMA-1^MET^ decreased relative cell number of GSCs irrespective of p53 status **A.** Western blotting analysis showing expression of MGMT, p53 and p21 in OPK111, OPK49, OPK161, 48EF and OPK257 GSCs. Actin was used as a loading control. **B.** Analysis of the cytotoxic effect of PRIMA-1^MET^ (10 or 20 μM) on OPK111, OPK49, OPK161, 48EF and OPK257 GSCs using trypan blue exclusion assay and automated cell counting to determine the percentage of relative number of cells in PRIMA-1^MET^-treated conditions relative to DMSO control at each time point (24 or 72 hours following initiation of a 24-hour treatment with PRIMA-1^MET^) (left) and the ratio of viable cells (% relative to total cell number in each experimental condition) (right) in the indicated cell lines. Data on graphs represent the mean values ± SD. **C.** Representative micrographs of OPK257 GSCs (original magnification 200X) treated with PRIMA-1^MET^ (10 or 20 μM) or DMSO control at 72-hour time point. Scale bar = 200 μm.

Given the potential role of GSCs in resistance to treatment and tumor relapse, we further investigated the effect of PRIMA-1^MET^ in GSCs maintained as neurosphere cultures. GSCs were derived from cancer specimens of patients with newly diagnosed GBM as previously described [58]. Western blotting analysis of MGMT protein levels showed that patient-derived GSCs OPK111, OPK161 and 48EF were MGMT-positive, while OPK49 and OPK257 were MGMT-negative. High expression of p53 protein with undetectable or very low levels of p21 evoked mutp53 status for OPK257 (Figure 8A). Prospective analysis of p53 by immunohistochemistry confirmed its strong expression in the corresponding patient pathology report (data not shown). Detection of very low levels of p53 protein and basal levels of p21 protein by Western blotting indicate that OPK111, OPK49, OPK161 and 48EF GSCs may display wtp53 function (Figure 8A).

We subsequently investigated whether PRIMA-1^MET^ exerts cytotoxic effects in the indicated GSCs. GSCs grown in complete stem cell culture medium were treated with PRIMA-1^MET^ or with vehicle DMSO control for 24 hours, then cells were re-suspended in drug-free stem cell culture medium for a total of 72 hours following PRIMA-1^MET^ or DMSO treatment initiation. We examined the relative cell number (percentage relative to DMSO control) and viable cell number (% relative to total cell number in each experimental condition) at 24 or 72-hour time points using trypan blue exclusion assay and automated cell counting. Exposure to PRIMA-1^MET^ for only 24 hours induced significant time and dose-dependent decrease in the relative cell number in all GSCs even after drug removal (Figure 8B and Table 4). At doses higher than 20 μM, PRIMA-1^MET^ caused massive cell death with the dominance of cellular debris.

**Table 4:** Relative cell number and viable cells (%) in GSC lines treated with a range of PRIMA-1^MET^doses

PRIMA-1^MET^, μM	24 hours		72 hours	
	Cell number, %^a^	p-value^b^	Cell number, %^a^	p-value^b^
	**OPK111**			
0	100±13.6	-	100±10.8	-
10	70.2±6.7	< 0.0001	61.1±7.7	< 0.0001
20	49.5±8.7	< 0.0001	29.4±4.9	< 0.0001
	**OPK49**			
0	100±8.8	-	100±5.8	-
10	62.4±7.2	< 0.0001	16.6±2.05	< 0.0001
20	49.97±5.4	< 0.0001	12.4±1.8	< 0.0001
	**OPK161**			
0	100±7.5	-	100±4.6	-
10	85.5±4.5	< 0.0001	64.6±3.6	< 0.0001
20	78.3±6.9	< 0.0001	40.1±4.2	< 0.0001
	**48EF**			
0	100±9.8	-	100±5.6	-
10	76.5±11.1	< 0.0001	59.95±5.6	< 0.0001
20	54.8±5.5	< 0.0001	8±1.6	< 0.0001
	**OPK257**			
0	100±8.3	-	100±10.8	-
10	104.4±6.2	n.s.	79.6±10	< 0.0001
20	75.1±5.2	< 0.0001	26.1±2.98	< 0.0001

PRIMA-1^MET^, μM	24 hours		72 hours	
	Viable cells,%^a^	p-value^b^	Viable cells, %^a^	p-value^b^
	**OPK111**			
0	84.1±5.4	-	95±2.9	-
10	84.6±4.2	n.s.	87±3.7	< 0.0001
20	83.4±4.9	n.s.	59.1±6.4	< 0.0001
	**OPK49**			
0	89±6.6	-	87.9±4.2	-
10	85.2±9.3	n.s.	71±4.9	< 0.0001
20	85.4±9.4	n.s.	62.6±4.4	< 0.0001
	**OPK161**			
0	92.2±6.8	-	92.8±5.3	-
10	92.8±7.3	n.s.	88.8±5.9	0.0006
20	91.4±6.6	n.s.	76.9±4.2	< 0.0001
	**48EF**			
0	90.2±8.4	-	91.4±5.5	-
10	87.8±3.9	n.s.	89.9±8.5	n.s.
20	88.1±3.8	n.s.	73.5±6.4	< 0.0001
	**OPK257**			
0	92.8±9.6	-	92.2±7.4	-
10	91.5±9.1	n.s.	88.1±9.9	0.039
20	43.7±7.3	< 0.0001	41.3±9.3	< 0.0001

a Mean ± SD

b Compared to DMSO control at the corresponding time point

PRIMA-1^MET^ at 20 μM did not induce significant decrease in cell viability (% of viable cells) in either MGMT-positive OPK111, OPK161 and 48EF or MGMT-negative OPK49 GSCs possessing wtp53 at 24 hours (Figure 8B and Table 4). However, at 72 hours after treatment with 20 μM their viability decreased significantly by 40.9±6.4%, 23.1±4.2%, 26.5±6.4% and 37.4±4.4%, respectively (p value < 0.0001). Similar dose induced 56.3±7.3% and 58.7±9.3% decrease in cell viability in mutp53 MGMT-negative OPK257 at 24 and 72 hours, respectively (p value < 0.0001). Of note, PRIMA-1^MET^ treatment for only 24 hours disrupted the morphology and structure of neurospheres in a dose-dependent manner, and abolished the formation of neurospheres (Figure 8C and Supplementary Figure S2).

The decrease in viable cell number at 72 hours following the initiation of treatment with 20 μM PRIMA-1^MET^ was also associated with a significant shift in average cell diameter from 12.78±3.3 μm to 11.96±3.4 μm in OPK111, from 14.04±3.9 μm to 10.96±4.3 μm in OPK49, from 14.31±2.94 μm to 12.67±4.96 μm in 48EF and from 15.44±3.6 μm to 11.32±6.0 μm in OPK257 (p value < 0.01), but not in OPK161 (Supplementary Figure S3).

Taken together, PRIMA-1^MET^ decreased relative cell numbers and disrupted the morphology and structure of neurospheres in a time- and dose- dependent manner in both MGMT-positive and –negative wtp53 GSCs at lower doses than in GBM established cell lines. In addition to the aforementioned effects, PRIMA-1^MET^ induced earlier and more pronounced effects on cell viability of mutp53/MGMT-negative GSC compared to other wtp53 GSCs.

### PRIMA-1^MET^ increased wtp53 and decreased mutp53 protein levels with concomitant decrease in MGMT protein levels and activation of Erk1/2 pathway in GSCs

**Figure 9:**
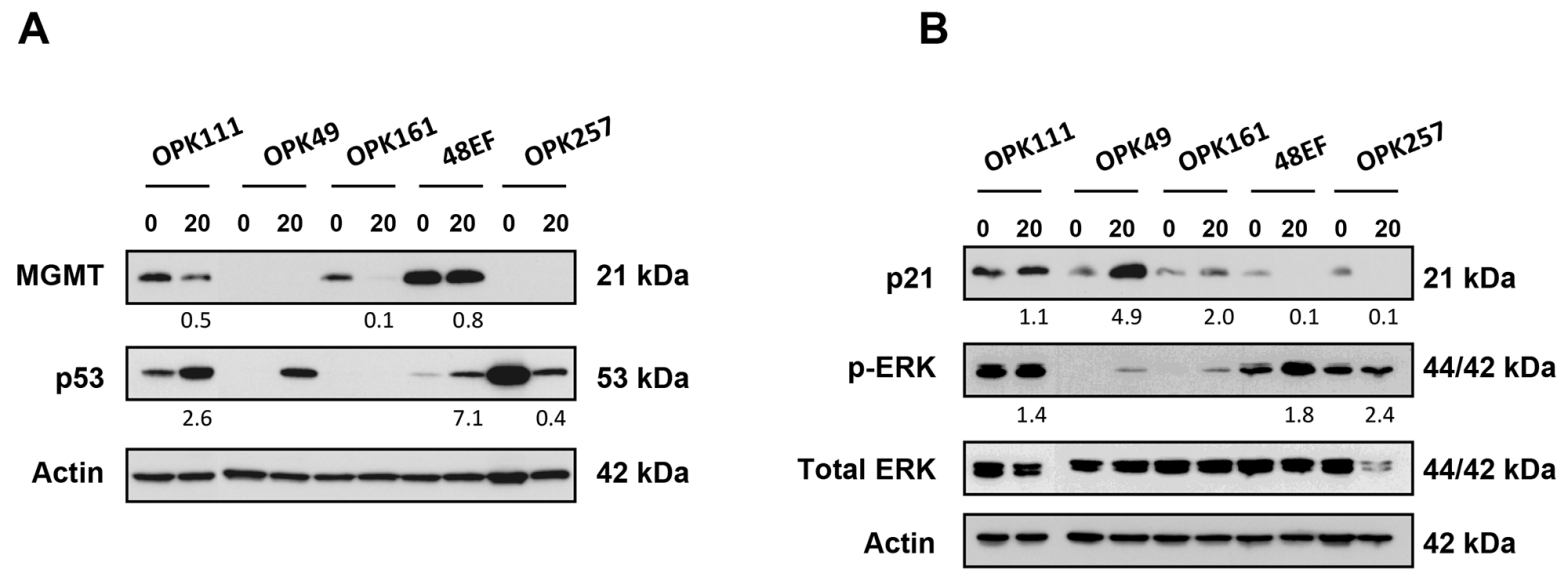
PRIMA-1^MET^ modulated expression of wt and mutp53, MGMT, p21 and phosphorylated forms of Erk1/2 in GSCs Western blotting analysis of expression of MGMT, p53 **(A)** p21 and phosphorylated forms of Erk1/2 (Thr202/Tyr204) **(B)** in OPK111, OPK49, OPK161, 48EF and OPK257 GSCs following 24-hour treatment with 20 μM PRIMA-1^MET^. Actin was used as a loading control. The density of the bands was normalized to that of DMSO controls (taken as 100%).

Next, to assess whether PRIMA-1^MET^ affects p53 and MGMT protein levels in GSCs, we analyzed by Western blotting total cellular protein of GSCs lysates following treatment with 20 μM PRIMA-1^MET^ or DMSO control for 24 hours. Interestingly, PRIMA-1^MET^ treatment increased p53 protein with concomitant decrease of MGMT protein levels, compared to DMSO control in wtp53 MGMT-positive OPK111 GSC (Figure 9A). There was no further increase of p21 protein (Figure 9B). PRIMA-1^MET^ induced a strong activation with increased p53 protein levels and approximately 5-fold increase of p21 protein in MGMT-negative OPK49 GSC. MGMT levels remained undetectable.

PRIMA-1^MET^ did not induce any changes in p53 protein levels, while MGMT levels were decreased in wtp53 MGMT-positive OPK161 GSC. PRIMA-1^MET^ induced activation of p53 without increase in p21 or significant changes in MGMT protein levels in MGMT-positive 48EF GSCs. In sharp contrast, PRIMA-1^MET^ treatment dramatically reduced mutp53 protein levels of MGMT-negative mutp53 OPK257 GSC line. We observed detectable levels of p21 expression in OPK257 treated with DMSO control, which could be mediated through p53-independent pathways. We did not detect caspase-3 or PARP-1 cleavage fragments by Western blotting in GSCs treated by PRIMA-1^MET^ 20 μM for 24 hours (data not shown).

Because PRIMA-1^MET^ treatment for 24 hours increased p-Erk1/2 in A172, T98/EV and T98/shRNA cell lines, we assessed whether PRIMA-1^MET^ induced similar effects in GSCs. Treatment with 20 μM PRIMA-1^MET^ for 24 hours increased Erk1/2 phosphorylation in all GSCs (Figure 9B) suggesting that Erk1/2 pathway was activated irrespective of p53 status or MGMT levels. Because of reduced cell number in all GSCs treated with PRIMA-1^MET^, we could not assess by Western blotting whether Erk1/2 activation was sustained in other time points beyond 24 hours of PRIMA-1^MET^ treatment.

## DISCUSSION

The intricate relationship between p53 and MGMT has not been investigated in light of recent studies highlighting the complex regulation of GOF mutp53 and its activities [20–22]. Because of the low number of GBM cell lines with available information about MGMT and mutp53 RPLA protein levels in the NCI-60 dataset, we investigated the causal relationship between MGMT and p53 using an isogenic pair of mut*TP53* expressing cells with at least 90% knockdown of MGMT. We showed that MGMT silencing decreased mutp53 at the protein level in T98G-based cell model. On the other hand, another study demonstrated that mutp53 knockdown in T98G cells decreased MGMT protein levels, suggesting that mutp53 contributes positively to MGMT expression [55]. Thus, a potential reciprocal positive relationship between mutp53 and MGMT may uphold the “mutp53/MGMT-positive” phenotype in this model known to harbor GOF mutp53 properties [20]. Previous studies showed that the abundance of mutp53 protein, a hallmark of p53 alterations in cancer, is required for GOF activities such as increased cell proliferation *in vitro* and *in vivo* [21, 59]. Several mechanisms might contribute to the regulation of mutp53 protein levels, such as increased half-life due to the lack of an auto-regulatory loop with the negative regulators MDM2 and MDMX [60], protection of *TP53* gene promoter against repressive histone modifications [61], microRNAs [62] and a transcriptional mechanism via histone deacetylase 8 [63]. Beyond its role as a DNA repair protein, MGMT interacts with >60 MGMT-binding proteins, including several histones and strongly binds to the heat-shock protein 90 (HSP90) [64] known to be involved in protection of mutp53 from ubiquitination [62, 65]. MGMT is also constitutively present at active transcription sites and co-precipitates with the transcription integrator CREB-binding protein CBP/p300 [66], which modulates nucleosomal histones and regulates p53 turnover [67]. The potential relationship between MGMT and mutp53 brings additional piece of evidence for the multifaceted role of MGMT in cancer [56, 66, 68].

We report a causal relationship between expression of MGMT and PRIMA-1^MET^-induced cytotoxicity through decreased levels of mutp53 protein without restoring wtp53 function in T98G-based model. We showed the convergence of several pathways underlying PRIMA-1^MET^-induced anti-proliferative and pro-apoptotic effects. Cell exposure to PRIMA-1^MET^ was associated with “loss” of G2 checkpoint and decrease in the S phase population in T98/shRNA. G2/M checkpoint prevents entry into mitosis and its abrogation in the context of MGMT silencing and mutp53 might be an indicator of abnormal response to DNA damage and a mitotic catastrophe, eventually leading to cell death [69]. Indeed, PRIMA-1^MET^ induced increased ratio of sub-G0/G1 apoptotic fraction and elevated levels of cleaved PARP-1 in T98/shRNA, indicating cell death through apoptosis. Increased susceptibility to apoptotic cell death has been reported in studies using siRNA-mediated knockdown of endogenous mutp53 in different cancer types [70–72]. PRIMA-1, the precursor compound of PRIMA-1^MET^ has been shown to induce nucleolar redistribution of mutp53 associated with p53 degradation *via* ubiquitination as a mechanism that removes the pro-survival function of mutp53 in a breast cancer model [73].

Treatment with PRIMA-1^MET^ increased expression of GADD45A protein in T98/shRNA, but not in T98/EV cells. This is in accordance with studies showing the selective role of GADD45A in the G2/M checkpoint and its function as a tumor suppressor protein through pro-apoptotic and growth suppression activities [74], possibly supported by a mechanism involving GADD45-induced inhibition of the kinase activity of the cdc2/cyclin B1 complex [75]. GADD45A is regulated in both p53-dependent and p53-independent manners. Interestingly, silencing of expression of mutp53 was shown to induce increased expression of wtp53-target genes including *GADD45A* in several human cell lines [70]. Decreased mutp53 levels in T98/shRNA cell line following treatment with PRIMA-1^MET^ could be involved in increased GADD45A.

Several lines of evidence suggest that PRIMA-1^MET^-induced cytotoxicity was not related to restoration of a wtp53 activity profile. Indeed PRIMA-1^MET^ failed to induce expression of wtp53-target genes, such as p21 for T98-based model. Using the antibody (PAb1620 [29]) that specifically recognizes wtp53 form, we found that PRIMA-1^MET^ did not promote proper folding of the mutant protein in immunofluorescence assays (data not shown). In a previous study using *in vitro* and *in vivo* models of primary and secondary GBM, functional p53-activating signals such as *CDKN2A* (p14^ARF^) were shown to be required for restoring p53 tumor-suppressor activities following treatment with PRIMA-1 [76]. This is in accordance with our finding showing that silencing of MGMT in T98G-based model harboring *CDKN2A* mutation and therefore lacking this important functional p53-activating signal, failed to restore wtp53 activity. Thus, restoring wtp53 function and induction of p53 target genes p21, MDM2, and GADD45A through a mechanism involving activation of wtp53 seems to be restricted to *CDKN2A* (p14^ARF^)-competent GBM cells, while selective induction of GADD45A could be achieved in the context of MGMT silencing and decreased expression of mutp53.

Sustained increased levels of phosphorylated Erk1/2 kinases up to 48 hours following treatment of T98/shRNA with PRIMA-1^MET^ is in accordance with a growing number of studies reporting implication of Erk1/2 in promoting cell death through apoptosis in different cancer types [77]. The role of Erk1/2 in apoptosis seems to be cell type-specific and also dependent on the levels of its expression, duration of its activity and subcellular localization [78]. The intensity and duration of pro- versus anti-apoptotic signals transmitted by Erk1/2 determines the cell fate towards proliferation or apoptosis. Cytosolic Erk1/2 restrains access to the transcription factor substrates and impedes survival and proliferative signals in the nucleus while increasing the catalytic activity of pro-apoptotic proteins such as death associated protein kinase (DAPK) in the cytoplasm [78].

PRIMA-1^MET^ decreased cell number and suppressed clonogenic capacity of mutp53 U138 cell line expressing intermediate MGMT protein levels to a greater extent compared to T98/EV and LN-18 cell lines. This may reflect recent findings showing the unequal effect of *TP53* mutations, with different mutants displaying a variable profile with respect to loss of wtp53 activity, the ability to inhibit wtp53, and the acquisition of GOF activities [21].

Further investigation of the effects of PRIMA-1^MET^ in established GBM cell lines showed that wtp53/MGMT-negative U87MG cell line displayed relatively strong basal levels of p21, heightened sensitivity to PRIMA-1^MET^, G1/M arrest and was the only cell line undergoing a senescent phenotype in response to PRIMA-1^MET^. Nonetheless, the senescent phenotype is potentially reversible in p53-intact cells, which may maintain the ability to re-proliferate and escape senescence [79]. By contrast, A172 (heterozygous SNP in p53 proline-rich domain) cell line was resistant to PRIMA-1^MET^. This could be related to pro-proliferative effects elicited by transient activation of Erk1/2. We also noted a dose-dependent increase of p21 expression without increased p53 levels, suggesting a p53-independent pathway for increased p21. High expression of p21 has been shown to contribute to resistance to drugs through anti-apoptotic effects [80] reported as an “antagonistic duality” of p21 through its role in inhibition of apoptosis [81].

Effects of PRIMA-1^MET^ in both wt and mutp53-harboring cells were reported in different types of cancer. A study conducted by Bao et al. [33] demonstrated that PRIMA-1^MET^ induced p53-dependent apoptotic cell death in wtp53 expressing malignant melanoma cells in 3D culture and in melanoma xenografts *in vivo*. The p53-dependent apoptosis was also triggered by PRIMA-1^MET^ in both mut and wtp53-harbouring Ewing sarcoma cells [82]. The concern that PRIMA-1^MET^ may likely bare toxicity risks for non-cancerous cells, associated with the effects of the drug observed in both wt and mutp53-harboring cells has been addressed in a previous study showing limited cytotoxicity toward normal hematopoietic cells, peripheral blood mononuclear cells and bone marrow mononuclear cells [83]. Potential p53-independent mechanisms of PRIMA-1^MET^-induced cell death involved reactive oxygen species (ROS) and other members of p53 family. PRIMA-1^MET^ toxicity in soft-tissue sarcoma cells was induced through a caspase-independent cell death. ROS-induced toxicity was associated with autophagy induction or JNK pathway activation [84]. Peng et al. [85] demonstrated that PRIMA-1^MET^ inhibited activity of thioredoxin reductase 1, an important regulator of cell redox balance, and thus, induced cell death through increased oxidation level in lung adenocarcinoma and osteosarcoma cells irrespective of p53 status. Moreover, PRIMA-1^MET^ was able to restore the pro-apoptotic function to mutp63 and p73 proteins sharing structural homology with p53, in the p53-null lung adenocarcinoma cells stably expressing temperature-sensitive mutant forms of these proteins [86].

To ascertain the potential clinical relevance for the use of PRIMA-1^MET^ in GBM, and because of the important role of GSCs as a disease reservoir in GBM [87], we used patient-derived GSCs with different levels of MGMT and p53 status. Surprisingly, PRIMA-1^MET^ exerted cytotoxic effects in all GSCs at lower concentrations than in established GBM cell lines. The most pronounced early effects on viability (24 hours) were seen in mutp53 MGMT-negative GSC line OPK257, similar to what we observed in T98/shRNA. This supports the general relevance of the effects described in T98/shRNA model and suggests that low levels of MGMT and decreased mutp53 levels correlate with increased cell sensitivity to PRIMA-1^MET^.

PRIMA-1^MET^ induced activation of wtp53, which was associated with decreased expression of MGMT in MGMT-positive GSCs OPK111. This is in accordance with previous studies showing that wtp53 down-modulates MGMT [22, 23], and a recent study showing that systemic delivery of wtp53 plasmid DNA using an immunoliposome nanocomplex to intracranial GBM tumors decreased MGMT and increased response of TMZ-resistant GBM tumors to TMZ in a mouse model [88]. Additional *in vitro* and *in vivo* studies to assess whether PRIMA-1^MET^ may sensitize TMZ-resistant GSCs through wtp53 activation and decreased expression of MGMT are warranted.

PRIMA-1^MET^ did not upregulate p53, while MGMT was downregulated in MGMT-positive wtp53 GSCs OPK161. This suggests that down-regulation of MGMT could be mediated by p53-independent mechanisms in GSCs. Perhaps, this could be mediated through the JNK pathway, which is critically involved in TMZ resistance and MGMT expression of MGMT-positive GSCs. Inhibition of JNK, either pharmacologically or by RNA interference in GSCs reduces their MGMT expression and alleviates TMZ resistance [89].

While induction of wild-type p53 protein by some cytotoxic agents often leads to growth arrest and subsequent apoptosis, PRIMA-1^MET^ did not induce PARP-1 or caspase-3 fragments cleavage in GSCs. All GSCs exhibited disruption of neurosphere morphology and structure, cell shrinkage and to some extent lysis of cells with cellular debris evoking necrotic cell death. A similar result was reported for other cell types. PRIMA-1, the precursor compound of PRIMA-1^MET^, induced necrosis with little apoptosis in mutp53 mouse leukemia L1210 cells [90].

In summary, we provide the first evidence for the convergence of PRIMA-1^MET^-induced molecular effects leading to activation of wtp53 associated with decreased MGMT protein expression in MGMT-positive GSCs or decreased mutp53 protein levels in mutp53/MGMT-negative cells (i.e., OPK257 and T98/shRNA).

Taken together, our results revealed a potential positive relationship between mutp53 and MGMT in T98G-based model and showed that silencing of MGMT sensitizes GBM cells possessing mut*TP53* to PRIMA-1^MET^-induced cell cycle arrest and apoptosis. Our findings underscore the cell-context dependent effects of PRIMA-1^MET^ in line with the wide diversity of mutp53 proteins [91] and the steadily evolving list of PRIMA-1^MET^ targets [84–86]. Our study further highlights that the final outcome and the cellular fate following PRIMA-1^MET^ treatment depend on MGMT protein levels and additional cell type-specific factors irrespective of p53 status: *i)* apoptosis in mutp53 GBM cells expressing very low levels of MGMT potentially mediated through abrogation of the G2 checkpoint control, activation of GADD45A and sustained expression of cytoplasmic phosphorylated Erk1/2 kinases (T98G-based model with MGMT silencing) and *ii)* senescence in MGMT-negative GBM cells harboring wtp53 (U87MG).

Future studies need to investigate the role of MGMT as a molecular target for sensitizing GBM cells to PRIMA-1^MET^ and whether PRIMA-1^MET^ may effectively sensitize GSCs to TMZ by decreasing MGMT protein levels. This will provide the proof-of-principle for the potential use of PRIMA-1^MET^ as a strategy to sensitize GSCs through pharmacological depletion of MGMT.

## MATERIALS AND METHODS

### Expression and mutation analysis of CCLE and NCI-60 cell lines

Normalized mRNA expression data (z-score values) for CCLE human cancer cell lines were extracted from the CCLE portal (available at http://www.broadinstitute.org/ccle) [40]. Data (log2 values) from reverse-phase protein lysate microarrays (RPLA) for NCI-60 panel of human cancer cell lines were extracted from CellMiner database (version 1.61) [52]. The information on *TP53* mutations in analyzed cell lines was obtained from the p53 website [41, 42], COSMIC [43, 44], and literature [45, 46]. SNB-19 glioma (derived from the same individual as U251 cell line [44]), SK-OV-3 ovarian (p53 mRNA and protein are undetectable [42]), OVCAR-5 ovarian (controversial p53 status), NCI-ADR-RES ovarian (similar to OVCAR-8 cell line), HL-60 leukemia (p53 null) [92], MDA-MB-435 and MDA-N melanoma (similar to M14 melanoma cell line [93]) cancer cell lines were excluded from the analyses of the NCI-60 and CCLE (SNB-19, SK-OV-3, MDA-MB-435, HL-60) datasets.

### Cell culture and drug treatment

The U87MG, T98G, A172, U138 and LN-18 GBM cell lines were obtained from American Type Culture Collection. T98G-based model described in [56] was used, where cells were transfected with plasmid vector encoding shRNA against MGMT (T98/shRNA) or with empty vector (T98/EV). The laboratory of Dr. Thierry Muanza (McGill University) kindly provided U87MG cells stably transfected with a plasmid carrying exogenous MGMT (U87/MGMT) or an empty vector (U87/EV) (transfection by Dr. Jad Ashami at the laboratory of Dr. Rolando Del Maestro). Established GBM cell lines were grown in Dulbecco's modified Eagle's medium (DMEM) supplemented with 10% fetal bovine serum (FBS; standard medium). GBM specimens used in this study were obtained from patients undergoing surgical treatment at the Montreal Neurological Hospital, in accordance with Institutional Review Board (IRB)-approved protocols. The diagnosis of GBM was made by a neuropathologist. GSCs isolated from cancer specimens were established and grown in neurosphere cultures as previously described [58]. GSCs expanded in neurosphere cultures retained self-renewal capacity in serum-free media, expressed neural stem cell markers, such as CD133 and nestin, and had the ability to differentiate in serum-containing growth media. 48EF GSCs were kindly provided by Dr. Samuel Weiss (University of Calgary). GSCs were maintained in neural stem cell complete medium NeuroCult NS-A Basal Medium with NeuroCult NS-A proliferation supplement (STEMCELL Technologies Inc., BC, Canada), Heparin (STEMCELL Technologies, BC, Canada), Epidermal Growth factor (EGF, 20 ng/ml) and Fibroblast Growth factor 2 (FGF-2, 20 ng/ml) (Life Technologies Inc., ON, Canada). All cell lines were grown at 37°C in a humidified atmosphere containing 5% CO_2_. Cells were treated with PRIMA-1^MET^ (Tocris Bioscience, Bristol, UK) dissolved in DMSO at varying doses in standard medium for 24 hours and then left in drug-free medium for additional time depending on the assay used. Cells treated with DMSO were used as a control.

### RNA isolation, PCR and sequencing

Total RNA was isolated from GBM cells using TRIzol^®^ reagent (Thermo Fisher Scientific Inc., Waltham, MA USA) according to the manufacturer's directions. The RNA was dissolved in 30 μl of DNase/RNase-free distilled water (Thermo Fisher Scientific Inc.). Reverse transcription was performed with 0.5 μg of total RNA using QuantiTect Reverse Transcription Kit (QIAGEN, Germantown, MD, USA) according to the manufacturer's directions. The regions corresponding to exons 3-4 (467 bp), exons 5-7 (498 bp) and exons 7-11 (532 bp) were amplified using the primers specific for sequences flanking each region. Amplification was performed in a 50 μl of a mixture containing AmpliTaq Gold 360 Master Mix (Thermo Fisher Scientific Inc.), 5 μl of cDNA and 0.5 μM of each primer. The amplification was carried out in a 2720 Thermal Cycler (Applied Biosystems) with an initial denaturation at 95°C for 5 min and followed by 35 cycles at 94°C for 15 s, 55°C for 1 min, 72°C for 1 min and a final extension for 10 min at 72°C. Amplicons were sequenced at the McGill University and Genome Quebec Innovation Centre using the same pairs of primers on an Applied Biosystems 3730xl DNA Analyzer (Sanger DNA sequencing).

**Table:** 

*TP53* gene exon	Forward (For) and reverse (Rev) primer sequences (5′→3′)
Exons 3-4	For: CAGTCAGATCCTAGCGTCGRev: CGGTAGATGTTCGTCAGT
Exons 5-7	For: CAGAAAACCTACCAGGGCRev: CCTGCCTTGTCGAAACTC
Exons 7-11	For: GACATAGTGTGGTGGTGRev: GAGGTGAAGAACAAGGGG

### Trypan blue exclusion cell viability assay

GBM cell cultures were subjected to varying doses of PRIMA-1^MET^ for 24 hours (24-hour time point) and then incubated for additional 24 (48-hour time point) or 48 hours (72-hour time point) in a drug-free medium. After that cells were washed with phosphate-buffered saline (PBS), trypsinized for 5 min and then neutralized by the addition of new complete medium. PBS used for washing was also collected to avoid losing easily detaching apoptotic cells (established GBM cell lines). Cells were pelleted by centrifugation at 1500 g for 10 min. The supernatant was aspirated and the cells were resuspended in a suitable volume of growth media (50-500 μl). The cell number and a ratio of dead cells with disrupted membranes (blue cells) to total number of cells was counted in triplicate for each well of plated cells using automated cell counter TC-10 (Bio-Rad Laboratories, Inc., Mississauga, ON, Canada) or automated Vi-CELL Cell Viability Analyzer (Beckman Coulter, Inc., Mississauga, ON, Canada). Cell number is represented as a percentage relative to cell number in control (100%). Percentage of viable (live) cells is represented in relation to the total cell number in each experimental condition.

### MTT assay

Cells were plated in 96-well plates at a density of 2500 cells per well in standard DMEM medium and allowed to adhere overnight at 37°C in 5% CO_2_. After that the cells were treated with PRIMA-1^MET^ at varying concentrations for 24 hours and left in drug-free medium for additional 24 hours before adding MTT. Cell proliferation was measured using Vybrant^®^ MTT Cell Proliferation Assay Kit (Thermo Fisher Scientific Inc.). 10 μl of 0.5% MTT 3-(4,5- dimethylthiazol-2-yl)-2,5-diphenyltetrazolium bromide was added to each well in the 96-well plates and 100 μl of 10% sodium dodecyl sulfate (SDS) was added 4 hours after adding MTT. After an overnight incubation, the absorbance was read at 570 nm.

### Clonogenic assay

Cells were plated in 6-well plates, allowed to adhere overnight and treated with PRIMA-1^MET^ at varying concentrations in standard medium for 24 hours. Then the medium was replaced with drug-free medium and the cells were incubated for additional 7-14 days or until colonies (more than 50 cells) were formed. Cells were then fixed with 10% formalin and stained using 1.5% methylene blue. Colonies of at least 50 cells were counted. The surviving fraction was normalized to the plating efficiency of the corresponding DMSO controls.

### Senescence assay

Cells were stained for senescence-associated beta-galactosidase activity (SA-β-Gal) as described by Dimri et al. [94] using Senescence β-Galactosidase Staining Kit (Cell signaling, Danvers, MA, USA) following the manufacturer's protocol. Briefly, cells were seeded in 6-well plate, allowed to adhere overnight, treated with PRIMA-1^MET^ at varying concentrations in standard medium for 24 hours, and left in drug-free medium for additional 120 hours (6 days after the start of treatment). Cells were then washed twice with PBS, fixed with 2% formaldehyde and 0.2% glutaraldehyde in PBS, and washed twice in PBS. Cells were stained for overnight in X-gal staining solution (1 mg/ml X-gal, 40 mmol/l citric acid/sodium phosphate (pH 6.0), 5 mmol/l potassium ferricyanide, 5 mmol/l potassium ferrocyanide, 150 mmol/l NaCl, 2 mmol/l MgCl_2_). Light microscopy was used to identify senescent (blue stained) cells. The percentage of SA-β-Gal positive cells was quantified by analyzing at least 400 cells in each experimental condition.

### Western blot analysis

Cells were washed twice (established cell lines) or collected (GSCs) with 1X cold PBS and lysed with 1X RIPA buffer (Boston BioProducts, Inc., Ashland, MA, USA) supplemented with 0.2 mM sodium orthovanadate, protease (Sigma-Aldrich, Oakville, ON, Canada) and phosphatase (Roche Diagnostics, QC, Canada) inhibitors cocktails. Proteins (30 μg, Pierce BCA protein assay kit, Thermo Fisher Scientific Inc.) were electrophoretically separated in 12% SDS-PAGE under reducing conditions and transferred onto PVDF membranes. Membranes were probed for MGMT (Santa Cruz, Dallas, TX, USA), p21^Waf/Cip1^ (Cell signaling, Beverly, MA, USA), mutant and wtp53 (DO-1, Santa Cruz), β-actin (Sigma-Aldrich, Oakville, ON, Canada), GADD45A (Abcam, Toronto, ON, Canada), cleaved PARP (D64E10, Cell signaling), phosphorylated Erk1/2 (Cell signaling), Erk1/2 (Cell signaling, Beverly, MA, USA) according to the manufacturer's recommendations. HRP activity was assayed by chemiluminescence using Amersham ECL Western Blotting Detection Reagent (GE Healthcare Life Sciences, Mississauga, ON, Canada). Quantitation of Western blot data was performed using ImageJ software analysis. All data were normalized to loading controls.

### Flow cytometry

Cells were treated with PRIMA-1^MET^ for 24 hours, collected, fixed in 70% ethanol, centrifuged, washed twice with PBS, and resuspended in 1 mg/ml RNase A (Sigma-Aldrich), incubated at 37°C for 30 minutes and suspended in 10 μg/ml propidium iodide working solution (Sigma-Aldrich) for 20 minutes at room temperature. Data were acquired on a BD FACSCanto II flow cytometer (Becton, Dickinson and Company, Franklin Lakes, NJ, USA) and analyzed with FlowJo (Version 9.6.2, FlowJo, LLC, Ashland, OR, USA) and ModFit LT (Verity Software House, Topsham, ME, USA) software.

### Immunofluorescence and confocal microscopy

For immunofluorescence staining, the cells were fixed with 4% paraformaldehyde for 10 min at room temperature, and then permeabilized with 100% methanol at −20 °C for 10 min. After blocking with 5% normal serum/ 0.3% Triton™ X-100 in PBS for 60 min at room temperature, cells were incubated with antibody against phospho-p44/42 MAPK (pErk1/2) (Thr202/Tyr204) (Cell signaling) at a working concentration of 1.44 μg/mL, diluted in 1% normal serum/ 0.3% Triton™ X-100 in PBS at 4 °C overnight, and then incubated with fluorescence-conjugated secondary antibody Alexa Fluor 488 (Life technologies) at a working concentration of 8 μg/mL diluted in antibody dilution buffer for 60 min at room temperature in the dark. Nuclei were stained with 0.1 μg/mL DAPI (Sigma). Images were captured (original magnification 400x) using a Zeiss LSM 780 laser scanning microscope (Carl Zeiss MicroImaging, Göttingen, Germany) and analyzed using ImageJ software (>40 cells analyzed in each experimental condition).

### Statistical analysis

We used GraphPad Prism (GraphPad Software Inc., La Jolla, CA, USA) to generate best-fit sigmoidal dose response curves for IC_50_ determination. Data are reported as mean +/− SD and are representative of at least 3 independent experiments unless otherwise stated. Statistics were performed using either an unpaired two-tailed Student's t-test or one-way ANOVA with a post-hoc test as appropriate. Correlations were estimated by Spearman's or Pearson's correlation methods. P values < 0.05 were considered statistically significant.

## SUPPLEMENTARY FIGURES AND TABLES








