# Supplementary material for: Sensitivity to PRIMA-1MET is associated with decreased MGMT in human glioblastoma cells and glioblastoma stem cells irrespective of p53 status
Source: Oncotarget. 2016 Aug 11;7(37):60245–69. doi: 10.18632/oncotarget.11197 (PMC5312382; doi:10.18632/oncotarget.11197)
Supplement: Supplementary file 1 [file oncotarget-07-60245-s001.pdf]

Sensitivity to PRIMA-1^{MET} is associated with decreased MGMT in human glioblastoma cells and glioblastoma stem cells irrespective of p53 status

SUPPLEMENTARY FIGURES AND TABLES

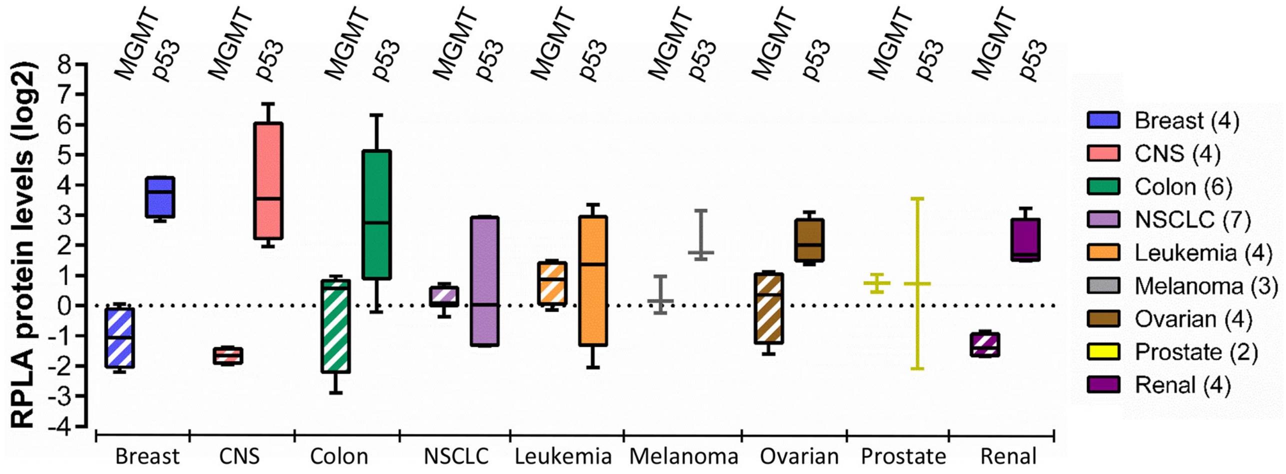

Protein	Primary site								
	Breast	CNS	Colon	NSCLC	Leukemia	Melanoma	Ovarian	Prostate	Renal
MGMT	-1.07	-1.65	0.58	0.09	0.875	0.16	0.365	0.745	-1.395
mutp53	3.78	3.555	2.745	0.04	1.375	1.75	2.02	0.73	1.69

Supplementary Figure S1: Correlation between mutp53 and MGMT protein levels in the NCI-60 cell line panel. Box and whisker plots (min-max, the horizontal line indicates median) of MGMT (dashed pattern fill) and p53 (solid fill) protein levels (from reverse-phase protein lysate microarrays, RPLA, log2) in NCI-60 cell lines with mutant *TP53* (n = 38) derived from 9 different cancer types. The median MGMT and p53 protein (RPLA) values for cell lines within each primary site are indicated in the table below the graph. CNS – central nervous system; NSCLC - Non-small cell lung cancer.

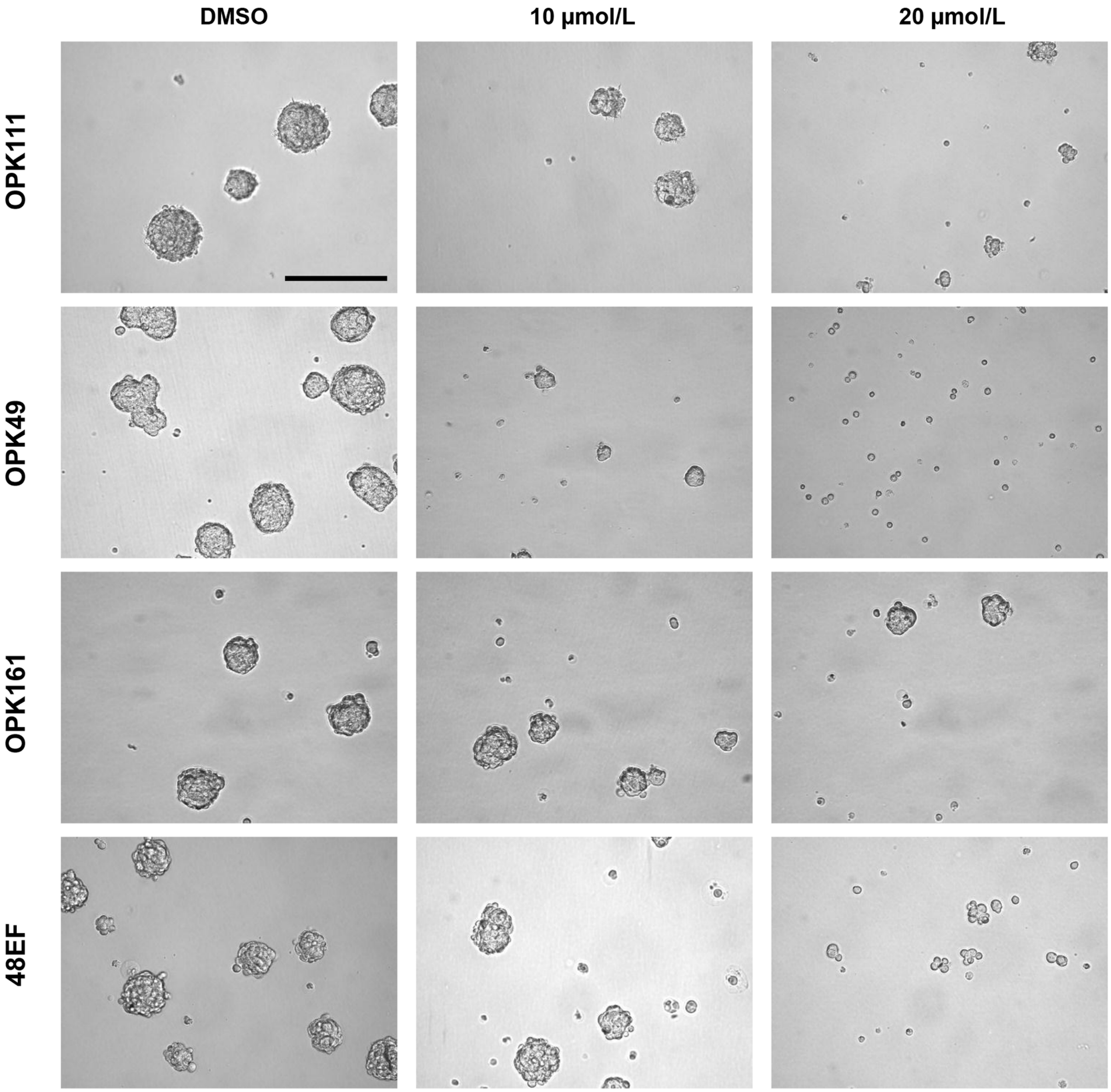

Supplementary Figure S2: Effect of PRIMA-1^{MET} on cell viability of GSCs. Representative micrographs of OPK111, OPK49, OPK161 and 48EF GSCs (original magnification 200X) treated with PRIMA-1^{MET} (10 or 20 µM) or DMSO control at 72-hour time point. Scale bar = 200 µm.

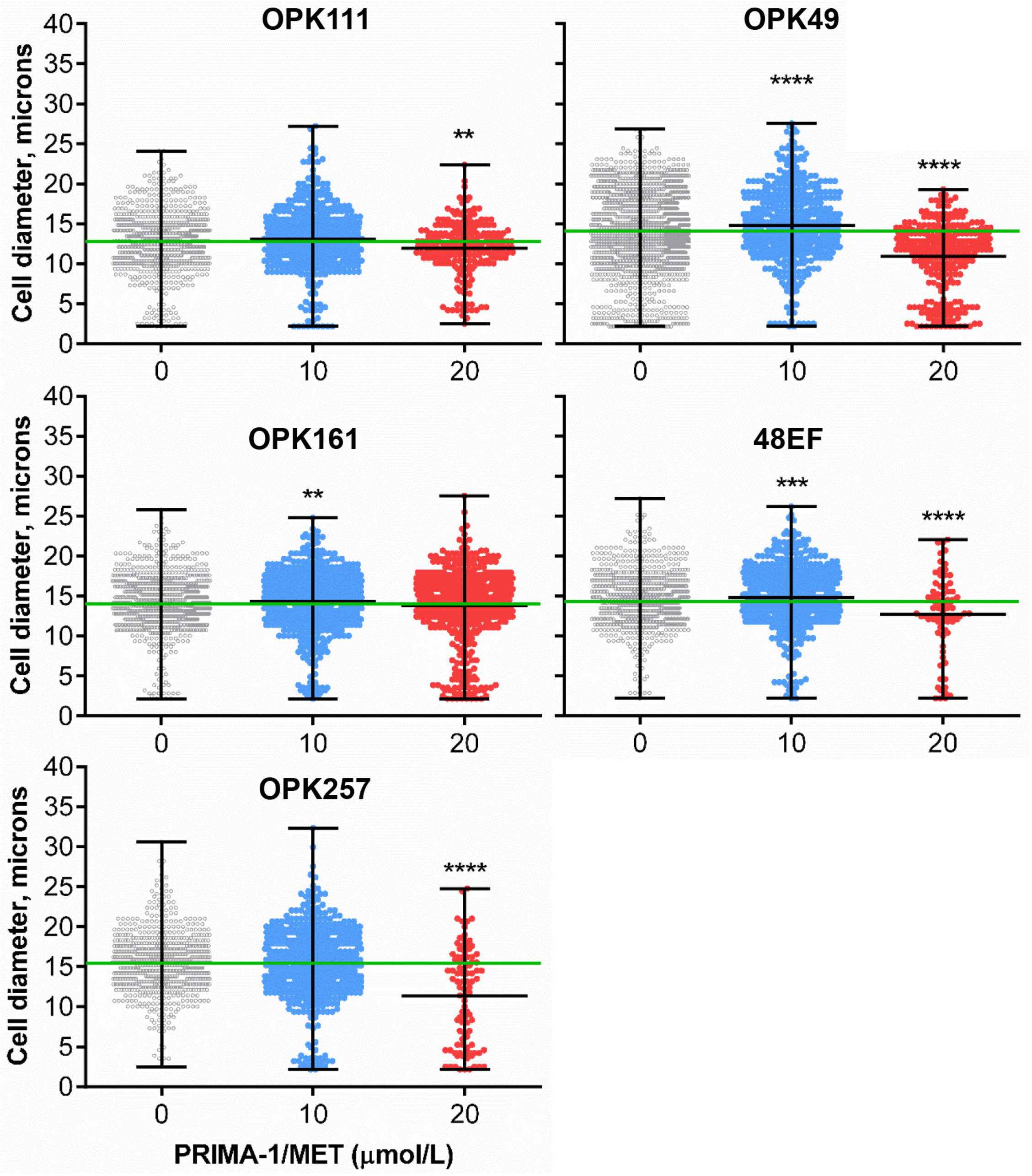

Supplementary Figure S3: Effect of PRIMA-1^{MET} on cell diameter of GSCs. Scatter plots (a range, individual horizontal lines indicate mean) of cell diameter in OPK111, OPK49, OPK161, 48EF and OPK257 GSCs treated with PRIMA-1^{MET} (10 or 20 μM) or DMSO control at 72-hour time point. The common horizontal line indicates mean cell diameter in DMSO control. *, statistically significant difference ($p < 0.05$) compared to DMSO control.

Supplementary Table S1: Normalized mRNA expression data (z-score values) of MGMT and p53 and *TP53* status in Cancer Cell Line Encyclopedia (CCLE) human cancer cell lines dataset.

See Supplementary File 1

Supplementary Table S2: MGMT and p53 protein levels (from reverse-phase protein lysate microarrays, RPLA) and *TP53* status in the NCI-60 cell lines panel. WT- wild-type, MT – mutant.

See Supplementary File 2

Supplementary Table S3: *TP53* (exons 3-11) sequence in T98/EV, T98/shRNA, U87MG and A172 GBM cell lines. The point mutations are indicated in bold red. Heterozygous single nucleotide polymorphism is specified with the alternate bases separated by a slash.

See Supplementary File 3
