# Supplementary material for: Sensitivity to PRIMA-1MET is associated with decreased MGMT in human glioblastoma cells and glioblastoma stem cells irrespective of p53 status
Source: Oncotarget. 2016 Aug 11;7(37):60245–69. doi: 10.18632/oncotarget.11197 (PMC5312382; doi:10.18632/oncotarget.11197)
Supplement: Supplementary file 4 [file oncotarget-07-60245-s004.docx]

**Supplementary table 3**.

| **U87MG** |
| --- |
| ACTTCCTGAAAACAACGTTCTGTCCCCCTTGCCGTCCCAAGCAATGGATGATTTGATGCTGTCCCCGGACGATATTGAACAATGGTTCACTGAAGACCCAGGTCCAGATGAAGCTCCCAGAATGCCAGAGGCTGCTCCCCGCGTGGCCCCTGCACCAGCAGCTCCTACACCGGCGGCCCCTGCACCAGCCCCCTCCTGGCCCCTGTCATCTTCTGTCCCTTCCCAGAAAACCTACCAGGGCAGCTACGGTTTCCGTCTGGGCTTCTTGCATTCTGGGACAGCCAAGTCTGTGACTTGCACGTACTCCCCTGCCCTCAACAAGATGTTTTGCCAACTGGCCAAGACCTGCCCTGTGCAGCTGTGGGTTGATTCCACACCCCCGCCCGGCACCCGCGTCCGCGCCATGGCCATCTACAAGCAGTCACAGCACATGACGGAGGTTGTGAGGCGCTGCCCCCACCATGAGCGCTGCTCAGATAGCGATGGTCTGGCCCCTCCTCAGCATCTTATCCGAGTGGAAGGAAATTTGCGTGTGGAGTATTTGGATGACAGAAACACTTTTCGACATAGTGTGGTGGTGCCCTATGAGCCGCCTGAGTTGGCTCTGACTGTACCACCATCCACTACAACTACATGTGTAACAGTTCCTGCATGGGCGGCATGAACCGGAGGCCCATCCTCACCATCATCACACTGGAAGACTCCAGTGGTAATCTACTGGGACGGAACAGCTTTGAGGTGCGTGTTTGTGCCTGTCCTGGGAGAGACCGGCGCACAGAGGAAGAGAATCTCCGCAAGAAAGGGGAGCCTCACCACGAGCTGCCCCCAGGGAGCACTAAGCGAGCACTGCCCAACAACACCAGCTCCTCTCCCCAGCCAAAGAAGAAACCACTGGATGGAGAATATTTCACCCTTCAGATCCGTGGGCGTGAGCGCTTCGAGATGTTCCGAGAGCTGAATGAGGCCTTGGAACTCAAGGATGCCCAGGCTGGGAAGGAGCCAGGGGGGAGCAGGGCTCACTCCAGCCACCTGAAGTCCAAAAAGGGTCAGTCTACCTCCCGCCATAAAAAACTCATGTTCAAGACAGAAGGGCCTGACTCAGACTG |
| **T98G** |
| ACTTCCTGAAAACAACGTTCTGTCCCCCTTGCCGTCCCAAGCAATGGATGATTTGATGCTGTCCCCGGACGATATTGAACAATGGTTCACTGAAGACCCAGGTCCAGATGAAGCTCCCAGAATGCCAGAGGCTGCTCCCCGCGTGGCCCCTGCACCAGCAGCTCCTACACCGGCGGCCCCTGCACCAGCCCCCTCCTGGCCCCTGTCATCTTCTGTCCCTTCCCAGAAAACCTACCAGGGCAGCTACGGTTTCCGTCTGGGCTTCTTGCATTCTGGGACAGCCAAGTCTGTGACTTGCACGTACTCCCCTGCCCTCAACAAGATGTTTTGCCAACTGGCCAAGACCTGCCCTGTGCAGCTGTGGGTTGATTCCACACCCCCGCCCGGCACCCGCGTCCGCGCCATGGCCATCTACAAGCAGTCACAGCACATGACGGAGGTTGTGAGGCGCTGCCCCCACCATGAGCGCTGCTCAGATAGCGATGGTCTGGCCCCTCCTCAGCATCTTATCCGAGTGGAAGGAAATTTGCGTGTGGAGTATTTGGATGACAGAAACACTTTTCGACATAGTGTGGTGGTGCCCTATGAGCCGCCTGAGTTGGCTCTGACTGTACCACCATCCACTACAACTACAT**A**TGTAACAGTTCCTGCATGGGCGGCATGAACCGGAGGCCCATCCTCACCATCATCACACTGGAAGACTCCAGTGGTAATCTACTGGGACGGAACAGCTTTGAGGTGCGTGTTTGTGCCTGTCCTGGGAGAGACCGGCGCACAGAGGAAGAGAATCTCCGCAAGAAAGGGGAGCCTCACCACGAGCTGCCCCCAGGGAGCACTAAGCGAGCACTGCCCAACAACACCAGCTCCTCTCCCCAGCCAAAGAAGAAACCACTGGATGGAGAATATTTCACCCTTCAGATCCGTGGGCGTGAGCGCTTCGAGATGTTCCGAGAGCTGAATGAGGCCTTGGAACTCAAGGATGCCCAGGCTGGGAAGGAGCCAGGGGGGAGCAGGGCTCACTCCAGCCACCTGAAGTCCAAAAAGGGTCAGTCTACCTCCCGCCATAAAAAACTCATGTTCAAGACAGAAGGGCCTGACTCAGACTG |
| **T98/EV** |
| ACTTCCTGAAAACAACGTTCTGTCCCCCTTGCCGTCCCAAGCAATGGATGATTTGATGCTGTCCCCGGACGATATTGAACAATGGTTCACTGAAGACCCAGGTCCAGATGAAGCTCCCAGAATGCCAGAGGCTGCTCCCCGCGTGGCCCCTGCACCAGCAGCTCCTACACCGGCGGCCCCTGCACCAGCCCCCTCCTGGCCCCTGTCATCTTCTGTCCCTTCCCAGAAAACCTACCAGGGCAGCTACGGTTTCCGTCTGGGCTTCTTGCATTCTGGGACAGCCAAGTCTGTGACTTGCACGTACTCCCCTGCCCTCAACAAGATGTTTTGCCAACTGGCCAAGACCTGCCCTGTGCAGCTGTGGGTTGATTCCACACCCCCGCCCGGCACCCGCGTCCGCGCCATGGCCATCTACAAGCAGTCACAGCACATGACGGAGGTTGTGAGGCGCTGCCCCCACCATGAGCGCTGCTCAGATAGCGATGGTCTGGCCCCTCCTCAGCATCTTATCCGAGTGGAAGGAAATTTGCGTGTGGAGTATTTGGATGACAGAAACACTTTTCGACATAGTGTGGTGGTGCCCTATGAGCCGCCTGAGTTGGCTCTGACTGTACCACCATCCACTACAACTACAT**A**TGTAACAGTTCCTGCATGGGCGGCATGAACCGGAGGCCCATCCTCACCATCATCACACTGGAAGACTCCAGTGGTAATCTACTGGGACGGAACAGCTTTGAGGTGCGTGTTTGTGCCTGTCCTGGGAGAGACCGGCGCACAGAGGAAGAGAATCTCCGCAAGAAAGGGGAGCCTCACCACGAGCTGCCCCCAGGGAGCACTAAGCGAGCACTGCCCAACAACACCAGCTCCTCTCCCCAGCCAAAGAAGAAACCACTGGATGGAGAATATTTCACCCTTCAGATCCGTGGGCGTGAGCGCTTCGAGATGTTCCGAGAGCTGAATGAGGCCTTGGAACTCAAGGATGCCCAGGCTGGGAAGGAGCCAGGGGGGAGCAGGGCTCACTCCAGCCACCTGAAGTCCAAAAAGGGTCAGTCTACCTCCCGCCATAAAAAACTCATGTTCAAGACAGAAGGGCCTGACTCAGACTG |
| **T98/shRNA** |
| ACTTCCTGAAAACAACGTTCTGTCCCCCTTGCCGTCCCAAGCAATGGATGATTTGATGCTGTCCCCGGACGATATTGAACAATGGTTCACTGAAGACCCAGGTCCAGATGAAGCTCCCAGAATGCCAGAGGCTGCTCCCCGCGTGGCCCCTGCACCAGCAGCTCCTACACCGGCGGCCCCTGCACCAGCCCCCTCCTGGCCCCTGTCATCTTCTGTCCCTTCCCAGAAAACCTACCAGGGCAGCTACGGTTTCCGTCTGGGCTTCTTGCATTCTGGGACAGCCAAGTCTGTGACTTGCACGTACTCCCCTGCCCTCAACAAGATGTTTTGCCAACTGGCCAAGACCTGCCCTGTGCAGCTGTGGGTTGATTCCACACCCCCGCCCGGCACCCGCGTCCGCGCCATGGCCATCTACAAGCAGTCACAGCACATGACGGAGGTTGTGAGGCGCTGCCCCCACCATGAGCGCTGCTCAGATAGCGATGGTCTGGCCCCTCCTCAGCATCTTATCCGAGTGGAAGGAAATTTGCGTGTGGAGTATTTGGATGACAGAAACACTTTTCGACATAGTGTGGTGGTGCCCTATGAGCCGCCTGAGTTGGCTCTGACTGTACCACCATCCACTACAACTACAT**A**TGTAACAGTTCCTGCATGGGCGGCATGAACCGGAGGCCCATCCTCACCATCATCACACTGGAAGACTCCAGTGGTAATCTACTGGGACGGAACAGCTTTGAGGTGCGTGTTTGTGCCTGTCCTGGGAGAGACCGGCGCACAGAGGAAGAGAATCTCCGCAAGAAAGGGGAGCCTCACCACGAGCTGCCCCCAGGGAGCACTAAGCGAGCACTGCCCAACAACACCAGCTCCTCTCCCCAGCCAAAGAAGAAACCACTGGATGGAGAATATTTCACCCTTCAGATCCGTGGGCGTGAGCGCTTCGAGATGTTCCGAGAGCTGAATGAGGCCTTGGAACTCAAGGATGCCCAGGCTGGGAAGGAGCCAGGGGGGAGCAGGGCTCACTCCAGCCACCTGAAGTCCAAAAAGGGTCAGTCTACCTCCCGCCATAAAAAACTCATGTTCAAGACAGAAGGGCCTGACTCAGACTG |
| **A172** |
| ACTTCCTGAAAACAACGTTCTGTCCCCCTTGCCGTCCCAAGCAATGGATGATTTGATGCTGTCCCCGGACGATATTGAACAATGGTTCACTGAAGACCCAGGTCCAGATGAAGCTCCCAGAATGCCAGAGGCTGCTCCCC**G/C**CGTGGCCCCTGCACCAGCAGCTCCTACACCGGCGGCCCCTGCACCAGCCCCCTCCTGGCCCCTGTCATCTTCTGTCCCTTCCCAGAAAACCTACCAGGGCAGCTACGGTTTCCGTCTGGGCTTCTTGCATTCTGGGACAGCCAAGTCTGTGACTTGCACGTACTCCCCTGCCCTCAACAAGATGTTTTGCCAACTGGCCAAGACCTGCCCTGTGCAGCTGTGGGTTGATTCCACACCCCCGCCCGGCACCCGCGTCCGCGCCATGGCCATCTACAAGCAGTCACAGCACATGACGGAGGTTGTGAGGCGCTGCCCCCACCATGAGCGCTGCTCAGATAGCGATGGTCTGGCCCCTCCTCAGCATCTTATCCGAGTGGAAGGAAATTTGCGTGTGGAGTATTTGGATGACAGAAACACTTTTCGACATAGTGTGGTGGTGCCCTATGAGCCGCCTGAGTTGGCTCTGACTGTACCACCATCCACTACAACTACATGTGTAACAGTTCCTGCATGGGCGGCATGAACCGGAGGCCCATCCTCACCATCATCACACTGGAAGACTCCAGTGGTAATCTACTGGGACGGAACAGCTTTGAGGTGCGTGTTTGTGCCTGTCCTGGGAGAGACCGGCGCACAGAGGAAGAGAATCTCCGCAAGAAAGGGGAGCCTCACCACGAGCTGCCCCCAGGGAGCACTAAGCGAGCACTGCCCAACAACACCAGCTCCTCTCCCCAGCCAAAGAAGAAACCACTGGATGGAGAATATTTCACCCTTCAGATCCGTGGGCGTGAGCGCTTCGAGATGTTCCGAGAGCTGAATGAGGCCTTGGAACTCAAGGATGCCCAGGCTGGGAAGGAGCCAGGGGGGAGCAGGGCTCACTCCAGCCACCTGAAGTCCAAAAAGGGTCAGTCTACCTCCCGCCATAAAAAACTCATGTTCAAGACAGAAGGGCCTGACTCAGACTG |
